# Densovirus Oil Suspension Significantly Improves the Efficacy and Duration of Larvicidal Activity against *Aedes albopictus*

**DOI:** 10.3390/v14030475

**Published:** 2022-02-25

**Authors:** Khadija Batool, Jie Xiao, Ye Xu, Ting Yang, Peiwen Tao, Siyu Zhao, Jiao Chen, Intikhab Alam, Yugu Xie, Jinbao Gu, Xiaoguang Chen

**Affiliations:** 1 Department of Pathogen Biology, Institute of Tropical Medicine, School of Public Health, Southern Medical University, Guangzhou 510515, China; khadijabatoolali@gmail.com (K.B.); xiaojie51@yahoo.com (J.X.); xuye266@gmail.com (Y.X.); zsy1252998112@outlook.com (S.Z.); gufii@i.smu.cn (Y.X.); gujinbao@smu.edu.cn (J.G.); 2 Wuhan Baile Health Technology Co., Ltd., Wuhan 430100, China; yangting56@yahoo.com (T.Y.); taopeiwen@webmail.hzau.edu.cn (P.T.); bgs@wdlz.com.cn (J.C.); 3 College of Life Sciences, South China Agricultural University, Guangzhou 510515, China; intikhabalam2013@gmail.com

**Keywords:** *Aedes albopictus*, densovirus, *Bacillus thuringiensis*, mosquito vector, larvicide, biological control

## Abstract

*Aedes albopictus* is the sole vector for various mosquito-borne viruses, including dengue, chikungunya, and Zika. Ecofriendly biological agents are required to reduce the spread of these mosquito-borne infections. Mosquito densoviruses (MDVs) are entomopathogenic mosquito-specific viruses, which can reduce the capacity of isolated vectors and decrease mosquito-borne viral disease transmission. However, their variable pathogenicity restricts their commercial use. In the present study, we developed a series of novel larvicide oil suspensions (denoted *Bacillus thuringiensis* (*Bti*) oil, *Ae. albopictus* densovirus (AalDV-5) oil, and a mixture of AalDV-5+*Bti* oil), which were tested against *Ae. albopictus* larvae under experimental semi-field and open-field conditions. The effect of AalDV-5 on non-target species was also evaluated. The combined effect of AalDV-5+*Bti* was greater than that of individual toxins and was longer lasting and more persistent compared with the laboratory AalDV-5 virus strain. The virus was quantified on a weekly basis by quantitative polymerase chain reaction (qPCR) and was persistently detected in rearing water as well as in dead larvae. Wildtype densovirus is not pathogenic to non-target organisms. The present findings confirm the improved effect of a mixed microbial suspension (AalDV-5+*Bti* oil) larvicide against *Ae. albopictus*. The development and testing of these products will enable better control of the vector mosquitoes.

## 1. Introduction

Mosquito-borne pathogens are serious health hazards and continue to affect human populations globally [1]. The *Aedes albopictus* mosquito is one of the most invasive mosquito species on the planet and is also a vector of several arboviruses of public health concern in the tropics and subtropics, including dengue, chikungunya, and Zika viruses [2,3,4]. Southern provinces of China such as Guangdong, Guangxi, Fujian, and Yunnan are particularly prone to dengue outbreaks because of their favorable environmental profile [5]. In Guangzhou, dengue fever can spread for more than seven months every year and it can spread during the hot summer months even in the northern temperate Beijing zone [6]. Long-term efforts to eradicate the vector have been hampered by the mosquito’s continuous migration and ability to quickly adapt to different environments [7]. The use of chemical insecticides is a leading approach for controlling vector-borne diseases. However, their widespread use has resulted in environmental pollution, insecticide resistance, and the need for new, long-lasting agents to control vector populations [8]. Our laboratory has previously reported the resistance of *Ae. albopictus* to several insecticides, including dichlorodiphenyltrichloroethane (DDT) and deltamethrin [9]. Mutations in two target sites, including knockdown resistance (*Kdr*) and the metabolic detoxification system, are responsible for insecticide resistance [9], and these mutations have also been observed in other mosquito species in many other places [10,11]. To reduce the present reliance on insecticide-based mosquito control, bio-control solutions aim to be long-term control agents and target a variety of mosquito species [12]. *Bacillus thuringiensis* (*Bti*) formulations [13,14], for example long-lasting *Ae. albopictus* larvicidal *Bti*-blocks, are highly effective in vector control strategies [15] and plant essential oils used for mosquito control [16,17,18].

Many viruses are pathogenic to mosquitoes [19,20], but their application in biological control has been limited due to their poor infectivity or difficult manufacturing processes that are not appropriate for field treatment. However, mosquito densoviruses (MDVs), which have a limited host range and various transmission patterns, are a possible alternative [21]. MDVs are highly mosquito-specific, do not infect unrelated organisms [22,23,24], and can replicate in numerous tissue types, such as the midgut, anal papillae, malpighian tubules, nerves, muscle fibers, fat bodies, and salivary glands, thus causing systemic infection [25]. *Ae. albopictus* densovirus was identified by our laboratory in wildtype *Ae. albopictus* sampled from dengue-endemic sites in Guangzhou, China, where infections of the first and second instar larvae of *Ae. aegypti*, *Ae. albopictus*, and *Culex quinquefasciatus* were found [26]. To improve the bio-efficacy of densovirus infection, we developed a novel biological larvicide, an oil suspension containing a mixture of *Ae*. *albopictus* densovirus (AalDV-5) and *Bti* oil toxins, and evaluated its effectiveness and persistence in a field-based setting.

## 2. Materials and Methods

### 2.1. Ethical Statement

The cell lines, including human endothelial cell line (HBMEC), were purchased from ScienCell (Carlsbad, CA, USA), and human glioma cells (U251), baby hamster kidney cells (BHK-21), *Drosophila melanogester* (S2), and *Spodoptera frugiperda* (Sf9) cell lines were bought from Sigma-Aldrich (St. Louis, MO, USA). African green monkey kidney (Vero) and fibroblast cell lines (Cos7) were bought from Thermo Fisher Scientific (Waltham, MA, USA). The *Ae. albopictus* C6/36 cell line was kindly provided by Professor Jingqiang Zhang’s laboratory, School of Life Science at Sun Yat-sen University (Guangzhou, China). The *Ae. aegypti*, Aag2 cell line was kindly provided by Professor Gong Chen’s laboratory, Medical School of Tsinghua University (Beijing, China). The specific growth mediums for cell lines were purchased from Thermo Fisher Scientific (Waltham, MA, USA). The cell lines BHK-21, HBMEC, Vero, and U251 were grown in DMEM medium added with 10% FBS at 37 °C. Sf9 cells were cultured in TC-100 medium supplemented with 10% FBS, at 37 °C with 5% CO_2_. Mosquito cell lines C6/36 and Aag2 were cultured in 1640 RPMI medium at 28 °C. The Drosophila S2 cell line was grown in Schneider’s Drosophila medium at 28 °C. The BALB/c suckling mice were obtained from the Animal Experiment Center of Southern Medical University, Guangdong Province, China. All animal experiments were performed according to guidelines established by the International Association for Evaluation and Acceleration of Laboratory Animal Care. Animal testing procedures were approved by the Office of Laboratory Animal Welfare (approval number: A5867-01) and animal care was performed according to institutional guidelines.

### 2.2. Mosquito Maintenance

*Ae. albopictus* (Foshan strain) was collected from Guangdong Province, China, and established in the laboratory. Briefly, mosquitoes were raised in a well-maintained insectary at 28 °C and 80% relative humidity, with a photoperiod of 12 h light and 12 h dark. Different stages of larvae were reared in stainless-steel trays (40 × 30 × 8 cm) filled with de-chlorinated tap water and fed regularly with turtle food (INCH-GOLD, Shenzhen, China). Adults were kept in BugDorm 30 × 30 × 30 cm cages, covered with a fine-net chiffon mesh, and fed with a 10% sucrose solution as the carbohydrate source. At five to seven days post-emergence, females were fed on mouse blood once per week. Moist filter papers were used to collect mosquito eggs after blood feeding. Bioassay experiments were performed with the first instar larvae of *Ae. albopictus*.

### 2.3. Study Design

The experimental period was from March to October 2020. We tested oil suspensions that had been stored for different times (0, 3, 6, and 9 months) to evaluate the efficacy of the product over time. Larval bioassays were conducted under experimental semi-field and open-field conditions. Bioassays were performed in plastic water containers. Viral titers in dead larvae and rearing water were evaluated. A pathogenicity analysis of AalDV-5 on non-target species, including in vitro cultured cells (mammalian and insects) and other animals, including carp, chickens, and mice, was performed.

### 2.4. AalDV-5 Formulation Testing

Microbial oil suspensions were developed by Wuhan Baile Health Technology Co. Ltd. (Wuhan, China). Methyl ester, denoted *methyl oleate* (9-octa-decenoic acid), was produced from methanol and oleic acid by esterification. Oleic acid and methanol were mixed and p-toluenesulfonic acid was added as the catalyst, and heated for reflux for 10 h, followed by cooling, neutralizing with sodium methoxide to a pH 8.5, washing with water to neutralize, drying with anhydrous calcium chloride (CaCl_2_), and performing vacuum distillation to obtain methyl oleate. The additives used in the oil suspension included castor oil polyoxyethylene ether, sodium dioctyl sulfosuccinate, polycarboxylate, and EO-PO block polyether, which make it water-soluble. The emulsified dispersions were thickened by adding hydrophobic organic soil and silica, which reduced cell clumps, sedimentation, and improved storage for long periods. The active components in the three test oil suspensions were: (i) *Bti* oil 20% *w*/*w* (potency: 1400 ITU/mg), (ii) *Ae. albopictus* densovirus-5 oil (Aa1DV-5) 0.1% *w*/*w* (10^9^ copies/mL), and (iii) mixed oil (AalDV-5+*Bti*). Commercially available *Bti* (7000 ITU/mg) was used in the oil suspension, while the AalDV-5 virus was obtained from our laboratory and collected according to a previous report [27]. Blank oil without microbes was used as a negative control. The three oil suspensions were tested at three-month storage intervals (March–October) to compare their toxicities. Oil suspensions were stored at 24 °C, until use.

### 2.5. Semi-Field Bioassay

**Figure 1:**
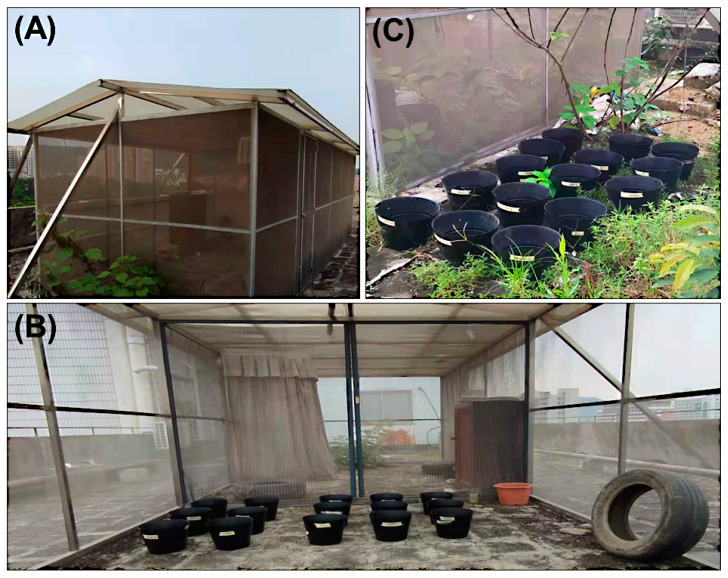
Bioassay environment. (**A**) Semi-field system (SFS). (**B**) Experimental semi-field bioassay treatment. (**C**) Experimental open-field bioassay treatment.

Semi-field trials were performed on the roof-top cage in a well-maintained rectangular semi-field system (SFS) [28]. The walls and roof of the semi-field hut were made of metal frames and fiberglass screens, and the roof was made of corrugated iron sheets (Figure 1A). In the experimental semi-field conditions, the water buckets were not directly exposed to sunlight, wind, or rain. As a result, water evaporation was minimal. The oil suspensions were mixed thoroughly, diluted 20 times in water, and stored as stock solutions at 4 °C. Viral concentrations were adjusted to 1 × 10 ^8^ copies/mL as the starting concentration using quantitative polymerase chain reaction (qPCR), as described before [27]. The AalDNV-F: AACCGATAGAACGAACAC, AalDNV-R: TTGGAGGACGACTGATTA primer pair was used for viral quantification. The laboratory strain AalDV-5 was also assessed under both experimental semi- and open-field conditions to further compare it with the oil suspensions. Semi-field bioassays were conducted with the first instar larvae (about 18 h after hatching) of *Ae. albopictus* in three biological replicates. First instar larvae were preferred for the bioassay as they are more vulnerable to both *Bti* and densovirus infection. Briefly, water buckets (15 cm height, 25 cm diameter) were filled with 4 L of water and batches of 25 first instar larvae were used per treatment (Figure 1B). Three buckets were used for each treatment. The larvae were infected with 1 × 10^8^ copies/mL of virus, using AalDV-5+*Bti* oil, AalDV-5 oil, or *Bti* oil. Food was not added to the test samples for 24 h post-exposure (pe). Thereafter, the larvae were provided with turtle food (INCH-GOLD, Shenzhen, China). When all larvae died in any test sample, the next batch of larvae was introduced. A water and blank oil treatment group were included as controls. Cumulative mortality rates, including dead larvae and pupae, were recorded daily for 32 days post-exposure (dpe). Dead larvae or pupae were not removed from the tested samples. The cumulative toxicity of each treatment was determined after every three-month interval under semi-field conditions. Each treatment was repeated in triplicate.

### 2.6. Open-Field Bioassay

Experimental open-field bioassays were performed in parallel with semi-field bioassays to determine the change in toxicity of the oil suspensions under different environmental conditions. The testing water buckets were kept in the open air on the roof of the public health department (Figure 1C), and other treatment parameters were similar to the semi-field experiments described above. Mortality was recorded daily.

### 2.7. Water Sampling and AalDV-5 Quantification

The viral titer in the larval rearing water was determined by qPCR analysis. To quantify the AalDV-5 genome copy number, a standard curve was created using a 10-fold serial dilution of a linear plasmid at known concentrations [29]. Water samples from both semi-field and open-field conditions were collected on a weekly basis, while lab strain AalDV-5 was sampled on a daily basis, and total genomic DNA was extracted using the MiniBEST Viral DNA Extraction Kit Ver. 5.0 (Takara, China) for qPCR. The primers used for viral detection and quantitation were based on a previous study by our laboratory [27]. The data were analyzed using Light Cycler 480 software (Roche, France).

### 2.8. Viral Detection in Dead Larvae

To determine the viral concentration in the dead larvae, single larvae were collected from all test samples, including semi-field, open-field, and lab-strain AalDV-5 samples. The total DNA was extracted. Each larva was homogenized in 100 μL of PBS buffer, and the homogenate was centrifuged for 5 min at 5000× *g* prior to DNA extraction. qPCR was performed as previously described [26].

### 2.9. Pathogenicity Assessment in Non-Target Species

An extensive pathological assessment of AalDV-5 was performed against non-target species, including in vitro cultured cells (mammals and insects) and other animal species, including mice, chickens, and carps. All species were exposed to wildtype AalDV-5, and total RNA and DNA were isolated and subjected to reverse transcription polymerase chain reaction (RT-PCR) and PCR to detect the relatively conserved, viral, non-structural (*NS*) protein gene. Each species had its own internal control (Table S1). All animal experiments were conducted with the approval of the Southern Medical University animal ethics board.

#### 2.9.1. Toxicity in Cultured Cells In Vitro

The toxicity of AalDV-5 against various non-target cell lines, including human brain micro-vascular endothelial cells (HBMEC), human glioma cells (U251), African green monkey kidney cells (Vero), African green monkey kidney fibroblast cells (Cos7), baby hamster kidney cells (BHK-21), *Drosophila melanogester* (S2), *Spodoptera frugiperda* (Sf9), and *Ae. aegypti* (Aag2) cells, was determined. Cells were infected with 200 uL of 1 × 10^11^ (copies/mL) AalDV-5 in cell culture flasks (25 cm^2^). A control group without the virus was included, and the cell growth status was monitored under a microscope. At four days post-exposure, cells were recovered, and total RNA was extracted using Trizol reagent according to the manufacturer’s instructions (Thermo Fisher scientific, Shanghai, China).

#### 2.9.2. Toxicity in Carp (Koi Carp)

To determine the toxicity of AalDV-5 against carp, each group was exposed to water containing AalDV-5 virus at a concentration of 1 × 10^11^ copies/mL for 30 min. The control group was exposed to virus-free tap water. This was repeated for two days, and morphological characteristics were observed and recorded each day. A pathological necropsy was performed at 30 days post-exposure according to a previously described method [30]. Different tissues were collected (including the heart, small intestine, swim bladder, and muscle tissue), and DNA and RNA were extracted.

#### 2.9.3. Toxicity in Poultry

Twelve broiler chickens were used for toxicity analysis of AalDV-5. The virus AalDV-5 (1 × 10^11^ copies/mL) was added to chicken drinking water. Chickens were exposed to the virus three times daily for three days, and thereafter were fed normally. The control group was exposed to virus-free water. The growth status of the chickens was observed daily, and a pathological necropsy was performed after 30 days according to a previously published method [31]. DNA and RNA were extracted from the different tissue samples for analysis by PCR and RT-PCR.

#### 2.9.4. Toxicity in Mice

A total of 12 Kunming mice at 6–8 weeks old were provided by the Animal Experiment Center of Southern Medical University. The virus AalDV-5 (1 × 10^11^ copies/mL) was added to the drinking water. Infected groups were exposed to the virus three times a day for three days, followed by normal feeding. A control group without viral exposure was included. The growth status of the mice was observed daily, and a pathological necropsy was performed at two weeks according to a previous publication [32]. Different tissues, including the small intestine, heart, liver, spleen, lung, kidney, and brain, were dissected and ground in liquid nitrogen, followed by DNA and RNA extraction.

#### 2.9.5. Acute Toxicity Assays with Mice (Acute Respiratory/Injection Pathogenicity Test)

A total of 16 Kunming mice (8 males and 8 females) at 6–8 weeks old were provided by the Animal Experiment Center of Southern Medical University. AalDV-5 infection (1 × 10^8^ copies/animal) was used in an acute respiratory/injection pathology test, and the volume of toxin in the respiratory tract did not exceed 0.3 mL/100 g body weight. Furthermore, the post-exposure effects of the virus in both assays were determined in male and female mice.

### 2.10. Statistical Analysis

Graphs were generated using GraphPad Prism 7 software. The error bars indicate the standard deviations from three independent biological replicates. The significance of the difference among the oil samples in different weeks was calculated by *t*-test using IBM SPSS version 22, and the significant difference is indicated by * (*p* < 0.05).

## 3. Results

### 3.1. Bioactivity of Oil Suspensions under Semi-Field Conditions

**Figure 2:**
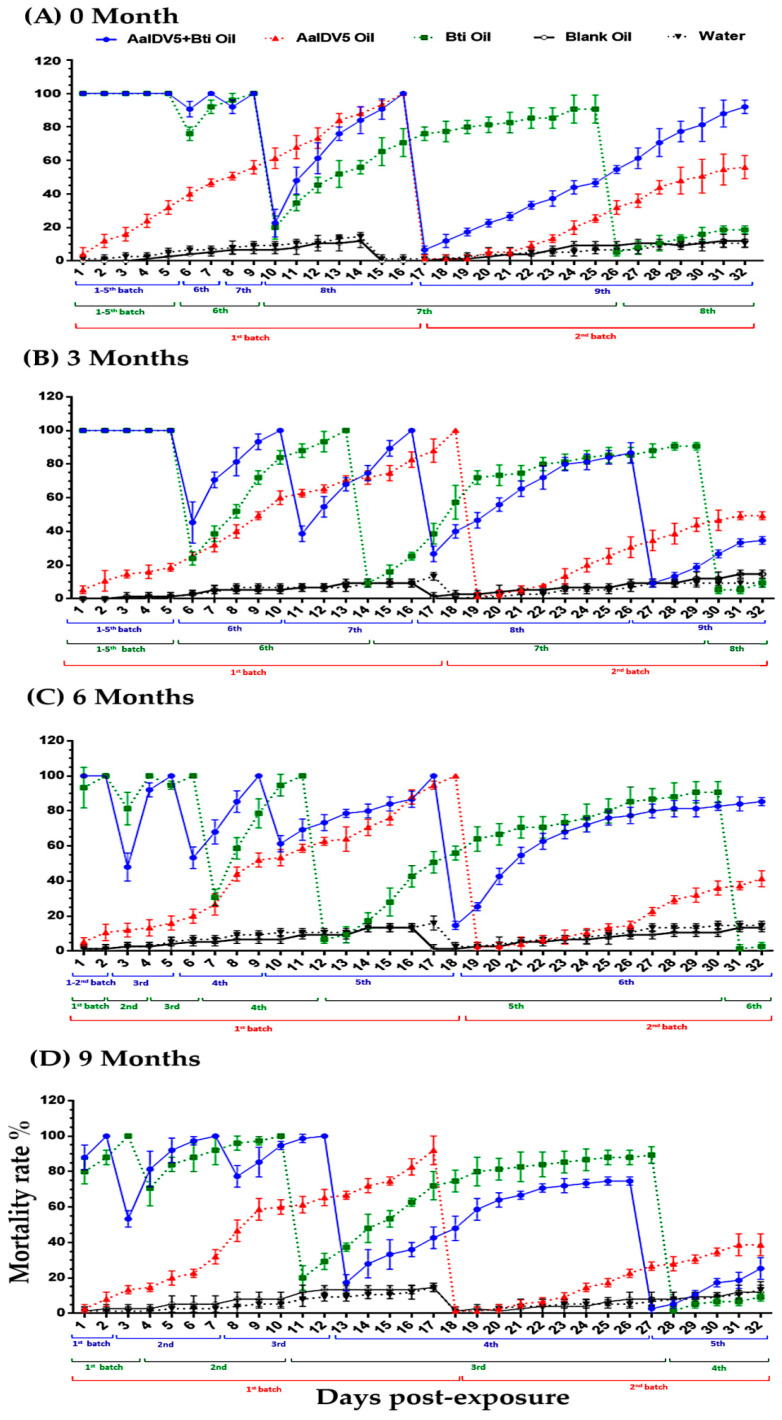
Comparative bioassay of oil suspensions over different storage times (0–9 months) against *Ae. albopictus* larvae in the semi-field condition. (**A**) Efficacy of oil suspensions on first instar larvae at 0 months, (**B**) 3 months, (**C**) 6 months, and (**D**) 9 months of storage. The bars indicate SDs from three independent (biological) replicates.

The comparative toxicities of three test oil suspensions, AalDV-5 oil, *Bti* oil, and a mixture comprised of AalDV-5+*Bti* oil, were examined in the first instar larvae of *Ae*. *albopictus* under semi-field conditions. In addition, their toxicity was assessed during storage at 3 months interval from 0 to 9 months (March–October). During the 4-week semi-field study of the 0-month sample, AalDV-5+*Bti* oil was applied to 9 batches of larvae that were added sequentially to water buckets, with 92% total mortality observed on day 32, whereas *Bti* oil was applied to 8 batches of larvae with 18.66% mortality. Two batches of larvae were added to the AalDV-5 oil group, and we observed a 56% mortality in the second batch on day 32 (Figure 2A and Figure S1A). The efficacy of the oil suspensions after 3 months of storage was then determined. Batch 9 larvae were added on day 27 to the AalDV-5+*Bti* samples, and the cumulative mortality was 34.66% on day 32, whereas 9.3% mortality in the eight batch was observed with *Bti* oil on day 32. The viral suspension, AalDV-5 oil, caused a 49.3% mortality rate in the second batch of larvae (Figure 2B and Figure S1B).

Toxicity analysis after a 6-month storage period showed that the AalDV-5+*Bti* oil, which was applied to 6 batches of larvae, resulted in 85.3% total mortality on day 32, but *Bti* oil resulted in 2.66% mortality in the sixth batch of larvae. The AalDV-5 oil resulted in 41% mortality in the second batch of larvae (Figure 2C and Figure S1C). The toxicity of the oil suspensions was also assessed after 9 months of storage, and the fifth batch of larvae, which were treated with AalDV-5+*Bti*, showed a 25.3% mortality rate on day 32. Conversely, a fourth batch of larvae was treated with *Bti* oil, and a 9.33% mortality rate was observed. The AalDV-5 oil pathogenicity analysis revealed 38.6% mortality in the second batch of larvae (Figure 2D and Figure S1D). Control groups, which were treated with water and blank oil, showed a 10–14% mortality rate in all testing groups and revealed that the blank oil (*methyl oleate*) had no toxicity, and toxicity was only induced by the active components of the oil suspensions. A previous study confirmed that *methyl oleate* is non-toxic to mosquito larvae (including *Ae. aegypti*, *Ae. albopictus*, and *Culex pipiens pallans*) [33].

The observed variation in the test suspension’s efficacy might be due to differences in its active components and mechanisms of action. In the 0–9-month samples, the efficacy of the mixed suspension (AalDV-5+*Bti*) was generally greater and longer lasting than that of AalDV-5 oil or *Bti* oil. The combination of both mosquito pathogens (bacterial and viral agents) may explain their improved activity under experimental semi-field conditions. Among different storage time samples, a slight decrease in efficacy was observed, but within the same group, the AalDV-5+*Bti* oil suspension was more effective.

### 3.2. Bioactivity of Oil Suspensions under Open-Field Conditions

**Figure 3:**
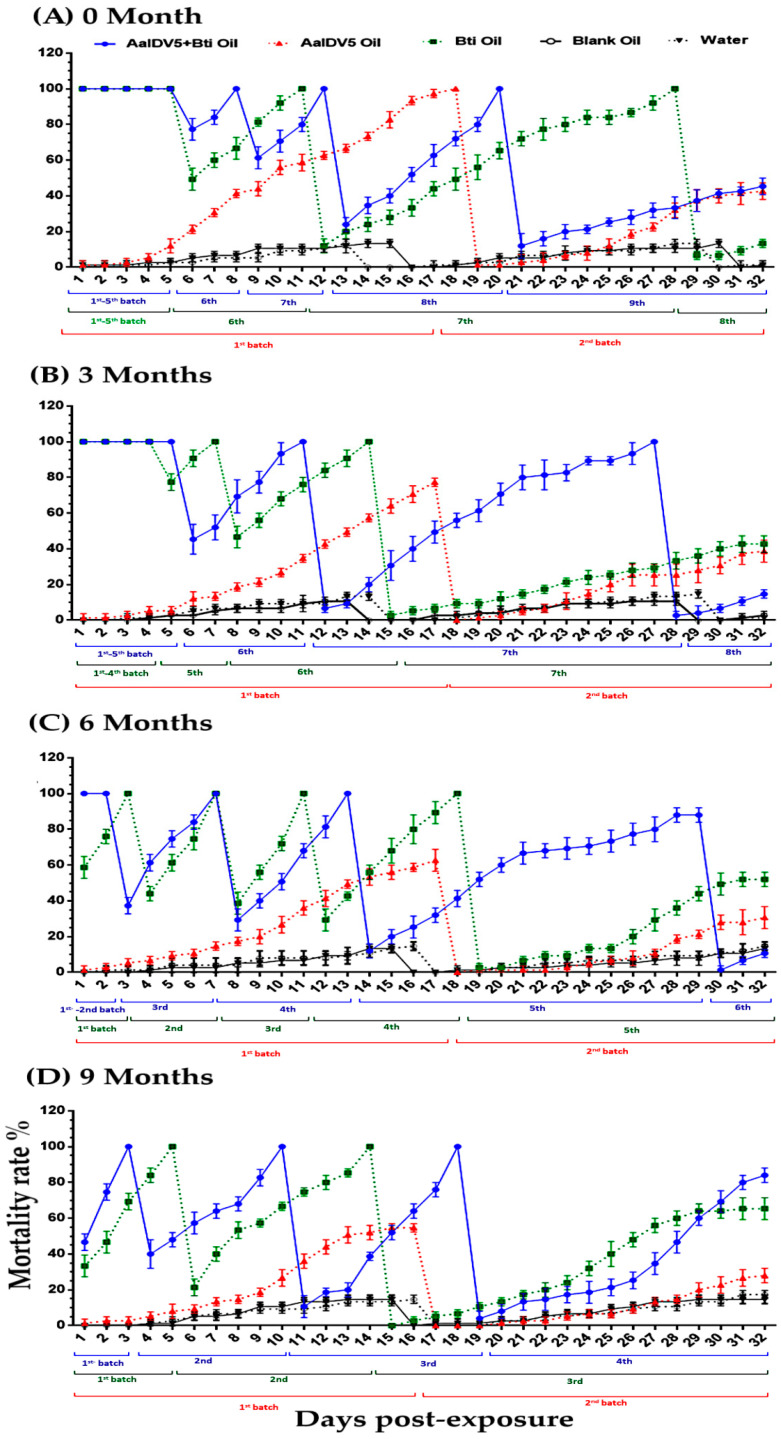
Comparative bioassay of oil suspensions over different storage times (0–9 months) against *Ae. albopictus* larvae in the open-field condition. (**A**) Efficacy of oil suspensions on first instar larvae at 0 months, (**B**) 3 months, (**C**) 6 months, and (**D**) 9 months of storage. The bars indicate SDs from three independent (biological) replicates, where each batch contains 25 larvae.

Experimental open-field trials in a small-scale area were conducted in water buckets to determine the efficacy and residual activity of oil suspensions. The efficiency of three test oil suspensions (AalDV-5+*Bti* oil, *Bti* oil, and AalDV-5 oil) was evaluated after each 3-month storage interval, up to 9 months. During the four-week open-field study of the 0-month group, we observed 100% mortality for the first five days for both mixed oil (AalDV-5+*Bti*) and *Bti* oil treatments (Figure 3A,B). Subsequently, the survival rate of larvae increased in the *Bti* oil treatment group compared with the AalDV-5+*Bti* oil treatment group. The AalDV-5+*Bti* oil-treated ninth batch of larvae had a 45.3% mortality rate on day 32, and we observed a 13.3% mortality rate in the eighth batch of larvae treated with *Bti* oil. AalDV-5 oil treatment of two batches of larvae resulted in a 42.6% mortality rate in the second batch (Figure 3A and Figure S2A). The 3-month test suspension resulted in a 100% mortality rate for the first five days in the AalDV-5+*Bti* treatment group and for four days in the *Bti* oil treatment group. Afterward, the mortality rate declined for seven batches of larvae treated with *Bti* oil, with 42.6% mortality observed, although eight batches of larvae were added to the AalDV-5+*Bti* treatment group and a 14.66% mortality rate was observed on day 32. AalDV-5 oil caused 38.6% total mortality in the second batch of larvae (Figure 3B and Figure S2B). After six months of storage, AalDV-5+*Bti* treatment caused 100% mortality for the first two days, and a total of six batches of larvae were treated with AalDV-5+*Bti*, resulting in a 10.66% mortality rate on day thirty-two. Five batches of larvae were treated with *Bti* oil, resulting in a 52% mortality rate. The activity of the only AalDV-5 oil samples decreased after three months. In the second batch of larvae treated with AalDV-5 oil, a 30.6% mortality rate was observed on day 32 (Figure 3C and Figure S2C). Toxicity assessments after 9 months revealed that that a total of 4 batches of larvae were added to AalDV-5+*Bti* oil samples with 84% mortality on day 32, whereas a reduction of *Bti* oil efficacy was observed for the 3 batches of larvae with 65.3% mortality. We observed a 28% mortality rate in the second batch of larvae treated with AalDV-5 oil (Figure 3D and Figure S2D). Greater than 84–86% survival was observed for both water and blank-oil control treatments in each group. The efficacy of the AalDV-5+*Bti* suspension lasts for longer than that of *Bti* oil or AalDV-5 oil alone in both semi-field and open-field environments. During the experimental period in semi- and open-field, the average temperature (March–October) ranged from 22 to 29 °C. The reduced efficacy of oil suspensions in experimental open-field environments may reflect the addition of dust particles and organic materials in the water buckets as a result of wind and rain, which may affect *Bti* and viral activity. Water evaporation due to sunlight exposure might be another factor that affects the overall stability of the microbes and their toxicity over time. These results indicate that the AalDV-5+*Bti* oil suspension mixture has potential for the development of an environmentally friendly, safe, and effective biological larvicide for *Ae. albopictus* control in the field.

### 3.3. Accumulation of the AalDV-5 in Larval Rearing Water

**Figure 4:**
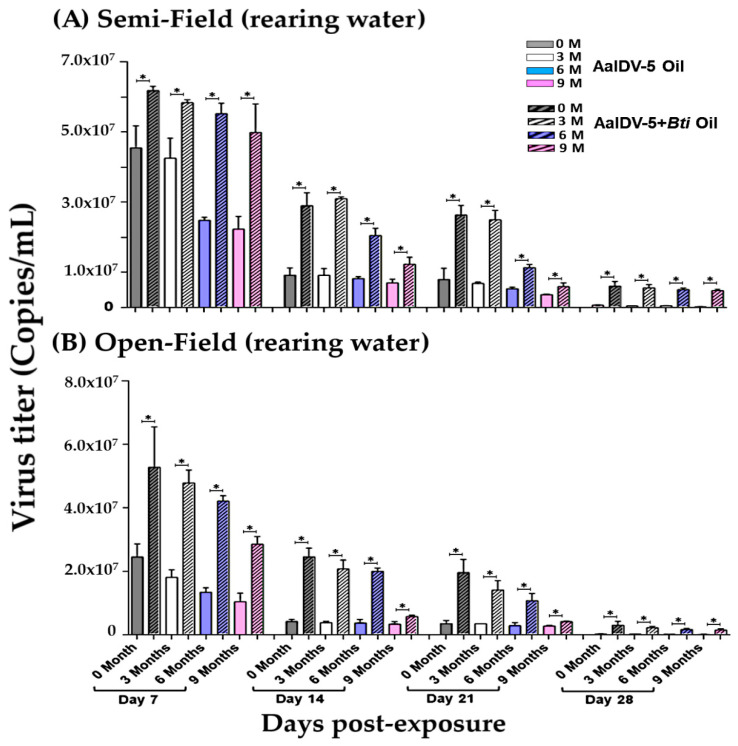
AalDV-5 quantification in larval rearing water. First instar *Ae. albopictus* larvae were exposed with AalDV-5 oil and AalDV-5+*Bti* oil (1 × 10^8^ copies/mL) in 4 L of water, and virus titer in rearing water was determined on a weekly basis in semi-field and open-field conditions. (**A**) AalDV-5 viral titer in semi-field conditions. Virus titer was determined on days 7, 14, 21, and 28. (**B**) AalDV-5 viral titer in open-field conditions. AalDV-5 titer was determined on days 7, 14, 21, and 28. The error bars indicate SDs from three independent (biological) replicates (*n* = 25 per replicate). The significance of the difference among the oil samples in different weeks was calculated by *t*-test using IBM SPSS (**A**,**B**), and the significant difference is indicated by * (*p* < 0.05).

First-instar larvae of *Ae. albopictus* were infected with a viral dose of 1 × 10^8^ copies/mL in a final volume of 4 L of water in both experimental semi- and open-field conditions. Once all the larvae died in any test sample, another group of 25 first instar larvae was placed into the bucket. Quantification of the AalDV-5 titer in larval rearing water exposed to AalDV-5+*Bti* and AalDV-5 oil suspensions was performed (Figure 4A,B, Tables S2 and S3). AalDV-5 was consistently detected in both semi- and open-field water samples (Figure 4A,B). The AalDV-5 titers in the larval rearing water exposed to AalDV-5+*Bti* oil (0–9 months) were significantly higher (*p* < 0.05) than those in the water exposed to only AalDV-5 oil from day 7 to day 28 post-exposure both in semi- and open-field conditions (Tables S2 and S3). Larval mortality was higher in the AalDV-5+*Bti* oil treatment groups as compared to only AalDV-5 oil in both semi- and open-fields. Storage time affected the overall efficacy of the oil suspensions. The viral titer was more stable in 0–3-month AalDV-5+*Bti* than in 6- or 9-month oil samples. The toxicity of the 6- and 9-month AalDV-5+*Bti* samples was relatively lower than that of the fresher suspensions, but was still significantly higher than that of the AalDV-5 oil suspension of 6 and 9 months. The decrease in viral titer observed during the second to fourth week may be related to different groups of new larvae entering the water bucket, larval food availability, and a decrease in water volume due to water evaporation, which could enhance the overall efficacy of the treatment. In both semi- and open-field conditions, environmental factors have the potential to influence viral replication. This could also be due to the virus being attached to the side of the bucket, the presence of larval food, or mosquito larvae taking up the virus [34].

### 3.4. Accumulation of AalDV-5 in Dead Larvae

**Figure 5:**
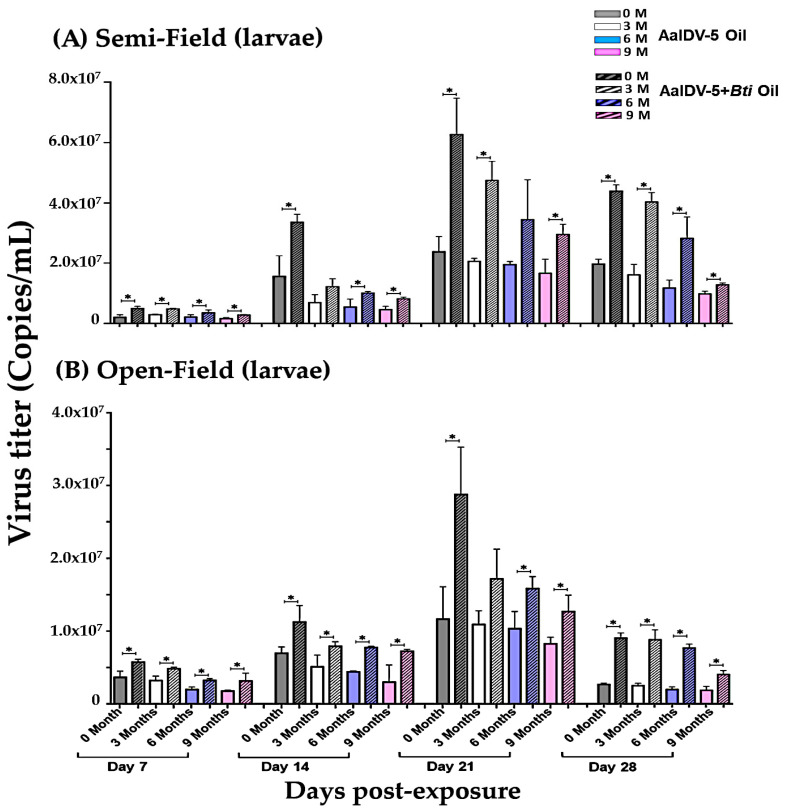
AalDV-5 quantification in larvae. First instar *Ae. albopictus* larvae were exposed with AalDV-5 oil and AalDV-5+*Bti* oil (1 × 10^8^ copies/mL) in 4 L of water, and virus titer in fourth instar single larvae was determined on a weekly basis in semi- and open-field conditions. (**A**) AalDV-5 viral titer in semi-field conditions. Virus titer was determined on days 7, 14, 21, and 28. (**B**) AalDV-5 viral titer in open-field conditions. AalDV-5 titer was determined on days 7, 14, 21, and 28, on a weekly basis. The error bars indicate SDs from three independent (biological) replicates (*n* = 25 per replicate). The significance of the difference among the oil samples in different weeks was calculated by *t*-test using IBM SPSS (**A**,**B**), and the significant difference is indicated by * (*p* < 0.05).

*Ae. albopictus* first instar larvae were exposed to AalDV-5+*Bti* and AalDV-5 oil suspensions. Viral concentrations of 1 × 10^8^ copies/mL were used in a final volume of 4 L of water in experimental semi- and open-field conditions. We added 25 larvae per batch to each treatment group. As before, a new batch of larvae was added to the bucket when all the larvae or pupae had died. Single dead larvae (fourth instar) were collected on a weekly basis and analyzed by qPCR for detection and quantification of AalDV-5 viral yields in both semi- and open-field environments (Figure 5A,B).

Viral titer was significantly higher in AalDV-5+*Bti* oil-treated larvae (0–9 months) from day 7 to day 28 compared to that in the AalDV-5 oil-treated larvae (0–9 months) in both semi- and open-field conditions (Tables S4 and S5). The lower viral titers observed under the experimental open-field conditions may be related to environmental factors such as sunlight, wind, and the presence of decaying leaves and soil particles in the water buckets. Among the AalDV-5+*Bti* oil suspensions that had been stored for different durations (0, 3, 6, and 9 months) both in semi- and open-field conditions, the 0-month (freshly prepared) and 3-month oil suspensions were more effective and persisted for longer than the 6- and 9-month suspensions. The viral titer decreased slightly with increasing storage time, but it was higher in the AalDV-5+*Bti* suspension-exposed larvae compared to in the AalDV-5 suspension-exposed larvae in both semi- and open-field conditions (Tables S4 and S5). Mixtures of different microbial pathogens may enhance the larvicidal action of the suspension and increase its stability over time compared to single toxin suspensions.

### 3.5. Bioactivity of Laboratory Strain AalDV-5 and Viral Quantification

**Figure 6:**
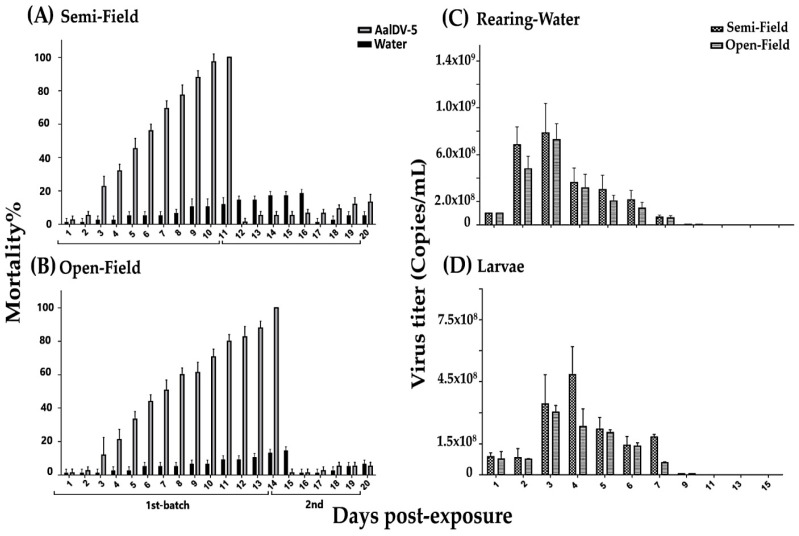
Bioassay of AalDV-5 against *Ae. albopictus* larvae. First instar larvae were treated with the AalDV-5 lab strain (1 × 10^8^ copies/mL) in (**A**) semi-field and (**B**) open-field environments; furthermore, the mortality rate of mosquito larvae were recorded for 20 days post-exposure with 3 biological replicates. The error bars indicate SDs from three biological replicates (*n* = 25 per replicate). (**C**) Virus quantification in larval rearing water and (**D**) larvae bodies in both semi-field and open-field conditions were evaluated (0–15 dpe), with the respective standard deviations from three biological replicates (*n* = 25 per replicate).

The larvicidal activity of the laboratory strain AalDV-5 was further assessed under both experimental semi- and open-field conditions. Three replicates of 25 first instar *Ae. albopictus* larvae were exposed to AalDV-5 (1 × 10^8^ copies/mL) in a final volume of 4 L of water. The semi-field bioassay resulted in 100% mortality in the first batch of larvae (Figure 6A). The activity of AalDV-5 quickly declined in the second batch of larvae, with 13.3% mortality observed on day 20 post-exposure. In the experimental open-field bioassay, 100% larval and pupal mortality occurred on day 14 post-exposure. In the second batch of larvae, the survival rate was higher, with 5% mortality on 20 dpe (Figure 6B). These results suggest that the residual activity of laboratory strain AalDV-5 was low in both semi- and open-field conditions compared to the oil suspension of AalDV-5. Furthermore, the AalDV-5 viral titer in the rearing water and larvae was quantified in both semi- and open-field environments (Figure 6C,D). The viral titers in both conditions were consistent (approximately 10^8^ copies/mL from 0–6 days post-exposure in the larval rearing water). Thereafter, the viral loads started to decline from day 7 (10^7^ copies/mL) and day 9 (10^6^ copies/mL) post-exposure. The lowest titer observed was 10 ^4^ copies/mL on days 13 and 15 post-exposure (Table S6). In addition, the viral titer in the larval bodies was quantified, and the maximum larval yield was at 4 dpe, with 4.84 × 10^8^ copies/larvae in the semi-field and 2.34 × 10^8^ copies/larvae in the open-field environment (Figure 6D, Table S6). Larval bodies had the highest viral yield of 10^8^ copies/larvae from 3 to 7 dpe. The viral titer dropped to 10^7^ at 7 dpe under open-field conditions. The lowest viral yield in the larval bodies was 10^4^ copies/larvae at 13–15 dpe under both semi- and open-field conditions (Table S6). The laboratory strain AalDV-5 displayed robust activity, which was not long-lasting compared with the oil suspension of AalDV-5. A decrease in viral concentration in the larvae exposed to the AalDV-5 lab strain may be linked with DNA extraction from different stages of larvae, which may correspond to varying DNA extraction efficiencies. Future studies should attempt to standardize DNA extraction across different larval stages, as well as using a parallel method to determine viral titer. On the other hand, water evaporation reduces the water volume and may affect viral replication. However, it is difficult to compare data from different studies because of differences in infection techniques, ambient conditions, viral titers, and stages of larval infection.

### 3.6. Safety Assessment of AalDV-5 on Non-Target Species

An extensive pathological assessment of AalDV-5 was performed on different species, including in vitro cultured cell lines, broiler chickens, mice, and carp (Figure S3, Table S7). *NS1* viral gene transcription was not detected in human cells HBMEC and U251, in monkey cells Vero and Cos-7, or in BHK-21 murine cells (Figure S3A-i). The *NS1* gene was also not detected in *Drosophila* S2 and *S. frugiperda* (Sf9) cells. However, *NS1* transcription was detected in *Ae. aegypti* (Aag2) cells, confirming the specificity of the virus. The internal organs of the control and test groups of carp (heart, small intestine, liver, gills, swim bladder, and muscle tissues), mice (heart, small intestine, liver, lung, kidney, spleen, and testis), and chickens were dissected. DNA and total RNA were extracted to confirm the expression of the conserved viral gene *NS1* after infection. *NS1* was not detected by PCR and RT-PCR (Figure S3) in the tissue of the inoculated or blank groups, indicating that there was no viral infection or replication. No acute respiratory toxicity was observed in any of the tested male or female mice nor was *NS1* detected in any of the acute-infected mice tissues (Figure S4, Tables S8 and S9). In contrast, *NS1* was detected in the infected positive control *Ae*. *albopictus* larvae, indicating that carp, chicken, and mice are non-target species for densovirus AalDV-5 infection (Table 1) and are not infected after ingesting the virus. Detailed results of each species tested were added to the Supplementary Materials.

Based on larval mortality and viral titer quantification in dead larvae and larval rearing water in experimental semi-field and open-field conditions, along with biosafety tests on non-target species, we showed that the mixture of densovirus and *Bti* oil suspension resulted in increased toxicity and improved bio-efficacy against *Ae. albopictus.* This product has excellent potential for application as a bio-control solution with minimal negative impact on the environment and the ability to effectively reduce the population of this mosquito vector, thus reducing the mosquito-borne diseases.

## 4. Discussion

MDVs are emerging as a promising tool for the control of *Aedes* mosquito populations. However, there are many limitations to the use of wildtype MDV insecticides because of their restricted commercial use. The major disadvantage of MDVs is their varying pathogenicity and slow activity, which tends to increase in a dose-dependent manner, depending on the viral titer and stage of infection [26,35]. MDVs have been isolated from many important mosquito species that are responsible for disease transmission, including *Ae. aegypti*, *Ae. albopictus*, *An. gambiae, An. sinensis*, *Cx. pipiens*, and *Cx. pipiens pallens* [25,26,36]. Larvicides are a key element of mosquito control, especially for targeting *Aedes* mosquitoes for integrated control and disease prevention. Natural organisms and their products have great potential as environmentally friendly tools for arthropod control and can be effectively used for vector control management [37].

In the present study, we developed a highly efficient mixed oil (AalDV-5+*Bti*) suspension larvicide under both experimental semi- and open-field conditions. The oil is composed of 80% methyl esters (*methyl oleate*), which were previously reported to be ineffective against mosquitoes, including *Ae. aegypti*, *Ae. albopictus*, and *Culex*. *pipiens pallens* [33]. The bioassays were carried out in plastic water containers that serve as *Aedes* breeding sites and were previously identified in a container survey for mosquito breeding sites [38]. The oil suspension protects the microbes against environmental conditions in experimental semi- and open-field settings, and the suspension had highly effective and long-lasting larvicidal activity against *Ae. albopictus* larvae. A previous semi-field study observed high larvicidal activity of bacterial formulations containing a mixture of bacterial insecticides (*Bactimos briquettes*) and two insect growth regulators (Altosid or Dudim) against *Ae. aegypti* [39]. Similarly, a binary mixture of a medicinal plant (*Carica papaya*) leaf extract and the bacterial toxin spinosad showed larvicidal and pupicidal activities against the mosquito *Ae. aegypti* under laboratory conditions [40]. The present semi-field study showed that the mixed larvicidal formulation AalDV-5+*Bti* provides long-lasting control of *Ae. albopictus* larvae in water buckets under semi- and open-field experimental conditions, compared with *Bti* oil or AlaDV-5 oil alone. Similar results were previously obtained with a combination of *Bti* and *Lysinibacillus sphaericus* (Lsph), which increased the residual activity against mosquitoes compared with the toxin components alone, probably due to the synergistic effects of the toxins [41]. Another previous study also described the combined synergistic effect of the herbicide glyphosate mixed with *L. sphaericus* against the third instar larvae of *Ae. aegypti* [42]. The active components in the test formulations have different modes of action. VectoBac *Bti* is a gut toxin that lyses and creates pores in the midgut epithelial cells of afflicted larvae [43], whereas MDV infection can occur in any larval tissue, including muscle fibers, the midgut, salivary glands, neurons, malpighian tubule, foregut, and hindgut [44]. Based on our findings, the combined effect of these microbes is that of a highly effective larvicidal agent.

*Bti* blocks were developed by our laboratory and have a high residual activity of at least 6 months against *Ae. albopictus* larvae in both the laboratory and open-field environments [15]. However, storage time affects the efficacy of the oil suspension. The toxicity of the suspensions decreased from 0 to 9 months, but the bioactivity of AalDV-5+*Bti* was significantly higher than that of the individual test suspensions in each group. The decrease in *Bti* oil efficacy is closely linked to abiotic factors in the open-field environment, such as contact with organic matter (leaf litter). *Bti* loses toxicity dramatically under open-field conditions, accounting for a portion of the observed loss in toxicity of the oil suspensions, which primarily affects bottom-feeding mosquitoes such as *Aedes* larvae [45]. Similarly, another study observed a decrease in the residual activity of *Bti* in a field environment, and the authors related the viability of *Bti* spores to the water temperature and the presence of a substrate. They determined that the toxicity remained stable in cold water for 21 days, whereas the toxicity of *Bti* in warm water decreased after just a few days [46].

Once mosquitoes become infected with mosquito-specific viruses, they are transferred to the aquatic environment during oviposition and replicate in the larval bodies. MDVs actively replicate in the larval bodies [47] and larvae shed viral particles through excretion in the aquatic environment [48]. The AalDV-5 viral titer in the AalDV-5+*Bti*-exposed larvae and larval rearing water remained high for a longer time than that of the AalDV-5 oil in both semi- and open-field conditions. Combining two insecticides can improve the treatment effectiveness. However, the viral titers under open-field conditions were lower than those under semi-field conditions for both the suspensions, but the larvicidal activity of AalDV-5+*Bti* oil was significantly higher than that of the AalDV-5 oil suspension. Under open-air treatment conditions, direct exposure to sunlight, rain, and organic matter considerably reduces the residual effectiveness of pesticides [49,50,51,52,53]. Temperature affects viral susceptibility, larval survival, lifespan, fertility, and fecundity, as well as other biological characteristics in insects [54,55,56,57,58]. This could also be due to the virus becoming attached to the sides of the bucket, or being absorbed by mosquito larvae [34]. We observed higher viral titers in the bodies of larvae treated with AlaDV-5+*Bti* compared to in the larval rearing water, suggesting that the virus was actively taken up by the larvae [47,59]. On the other hand, *Bti* is a gut poison producing many kinds of insecticidal proteins during its growth phase [60]. *Bti* facilitates gut damage, favoring MDV infection in AlaDV-5+*Bti* oil suspension. The bioactivity of the oil suspensions and laboratory strain AalDV-5 was observed for four weeks, and the laboratory strain AalDV-5 could not be detected after some time, unlike the AalDV-5 or AalDV-5+*Bti* oil suspensions. The oil therefore protected the microbes for an extended period of time, which is a promising finding for the application of this larvicidal agent.

Combinations of different pathogens in mixed oil may have synergistic interactions that improve the bioactivity of the oil suspension. Safety assessments of AalDV-5 revealed the absence of toxicity in all tested species. MDVs are highly host-specific and not infectious to unrelated organisms, such as bees, butterflies, crustaceans, or worms, as well as birds, fish, rats, rabbits, hamsters, or other mammals [22,23,61,62,63]. In addition, as densovirus is non-pathogenic towards mammals, there are no concerns about public safety and health issues in humans. These characteristics make the densovirus research an eco-friendly and safe mode for pest control and management. However, the toxicity of oil suspensions in other mosquito species and in the natural environment is yet to be determined. The development and testing of these products will help to reduce the population of vector mosquitoes and provide a theoretical basis for further studies to improve the toxicity and decrease the larval resistance for more effective vector control strategies.

## 5. Conclusions

Our findings shed light on the importance of using microbial toxin mixtures (AalDV-5+*Bti*) for improved efficacy against mosquitoes. The mixed oil (AalDV-5+*Bti*) suspension showed good residual efficiency for *Ae. albopictus* larvae in widely used plastic water container habitats in both experimental semi- and open-field trials. The oil form protects microbes in mixed samples for a long time and maintains bioactivity over time. Furthermore, the virus titer was found to be more persistent and long-lasting in mixed oil suspensions as compared to the AalDV-5 oil and laboratory strain. The dual approach in the mixed oil (AalDV-5+*Bti*) could be useful for mosquito control as well as for elucidating future vector control and management directions. It is possible that a single application of mixed oil suspensions in mosquito breeding sites such as ditches, ponds, and artificial containers could provide long-term effective control of *Ae. albopictus*. Long-term follow-up trials in a large-scale natural environment are required to determine the long-term consequences of such formulations. Future studies are needed to elucidate the activity of such products in other mosquito species in natural habitats.

## Figures and Tables

**Table 1:** The infection and pathogenicity of AalDV-5 on non-target species.

Type	Test Species	AalDV-5 Concentration Used (Copies/mL)	Application	PCR Detection	Pathological Changes	Target Species	Non-Target Species
Mammal	Mice	1 × 10^8^	Respiratory toxicity	Not detected	Not observed	_	+
	Mice	1 × 10^8^	Injection	_	_	_	+
		1 × 10^11^	Oral	_	_	_	+
Bird	Chicken	1 × 10^11^	Oral	_	_	_	+
Aquatic	Carp	1 × 10^11^	Oral	_	_	_	+
Cell lines	HBMEC	1 × 10^11^	Inoculation	_	_	_	+
	U251	1 × 10^11^	Inoculation	_	_	_	+
	Vero	1 × 10^11^	Inoculation	_	_	_	+
	Cos-7	1 × 10^11^	Inoculation	_	_	_	+
	BHK-21	1 × 10^11^	Inoculation	_	_	_	+
	S2	1 × 10^11^	Inoculation	_	_	_	+
	Sf9	1 × 10^11^	Inoculation	_	_	_	+
	C6/36	1 × 10^11^	Inoculation	+	+	+	_
	Aag2	1 × 10^11^	Inoculation	+	+	+	_

## Data Availability

The datasets that support the conclusions of this article are included in this article and Supplementary Files.

## Supplementary Materials

The following supporting information can be downloaded at: https://www.mdpi.com/article/10.3390/v14030475/s1, Figure S1: Rate of pupation in the semi-field bioassay. Figure S2: Rate of pupation in an open-field bioassay. Figure S3: Effect of AalDV-5 on non-target species. Figure S4: Acute respiratory toxicity and injection pathogenicity test in male/female mice. Table S1: The internal control used for RT-PCR and PCR. Table S2 AalDV-5 titer in the larval rearing water in semi-field environment. Table S3: AalDV-5 titer in the larval rearing water in open-field environment. Table S4: The AalDV-5 viral accumulation in larvae in semi-field condition. Table S5: The AalDV-5 viral accumulation in larvae in open-field condition. Table S6: Lab-strain AalDV-5 virus titer in larval rearing water and larvae body. Table S7: Effect of AalDV-5 on non-target species. Table S8: Acute respiratory toxicity test in mice. Table S9: Acute injection toxicity test in mice.

## References

[1] Stanaway J.D., Shepard D.S., Undurraga E.A., Halasa Y.A., Coffeng L.E., Brady O.J., Hay S.I., Bedi N., Bensenor I.M., Castañeda-Orjuela C.A. (2016). The global burden of dengue: An analysis from the Global Burden of Disease Study 2013. Lancet Infect. Dis..

[2] Lwande O.W., Obanda V., Lindström A., Ahlm C., Evander M., Näslund J., Bucht G. (2020). Globe-trotting *Aedes aegypti* and *Aedes albopictus: Risk* factors for arbovirus pandemics. Vector Borne Zoonotic Dis..

[3] Cuthbert R.N., Diagne C., Haubrock P.J., Turbelin A.J., Courchamp F. (2021). Are the “100 of the world’s worst” invasive species also the costliest?. Biol. Invasions.

[4] Lowe S., Browne M., Boudjelas S., De Poorter M. (2000). 100 of the World’s Worst Invasive Alien Species: A Selection from the Global Invasive Species Database.

[5] Liu K., Hou X., Ren Z., Lowe R., Wang Y., Li R., Liu X., Sun J., Lu L., Song X. (2020). Climate factors and the East Asian summer monsoon may drive large outbreaks of dengue in China. Environ. Res..

[6] Metelmann S., Liu X., Lu L., Caminade C., Liu K., Cao L., Medlock J.M., Baylis M., Morse A.P., Liu Q. (2021). Assessing the suitability for *Aedes albopictus* and dengue transmission risk in China with a delay differential equation model. PLoS Negl. Trop. Dis..

[7] Mayer S.V., Tesh R.B., Vasilakis N. (2017). The emergence of arthropod-borne viral diseases: A global prospective on dengue, chikungunya and Zika fevers. Acta Trop..

[8] Weill M., Lutfalla G., Mogensen K., Chandre F., Berthomieu A., Berticat C., Pasteur N., Philips A., Fort P., Raymond M. (2003). Insecticide resistance in mosquito vectors. Nature.

[9] Li Y., Xu J., Zhong D., Zhang H., Yang W., Zhou G., Su X., Wu Y., Wu K., Cai S. (2018). Evidence for multiple-insecticide resistance in urban *Aedes albopictus* populations in southern China. Parasites Vectors.

[10] Li Y., Zhou G., Zhong D., Wang X., Hemming-Schroeder E., David R.E., Lee M.C., Zhong S., Yi G., Liu Z. (2021). Widespread multiple insecticide resistance in the major dengue vector *Aedes albopictus* in Hainan Province, China. Pest Manag. Sci..

[11] Cisse M.B., Keita C., Dicko A., Dengela D., Coleman J., Lucas B., Mihigo J., Sadou A., Belemvire A., George K. (2015). Characterizing the insecticide resistance of *Anopheles gambiae* in Mali. Malar. J..

[12] Benelli G., Jeffries C.L., Walker T. (2016). Biological control of mosquito vectors: Past, present, and future. Insects.

[13] Johnson B.J., Manby R., Devine G.J. (2020). Performance of an aerially applied liquid *Bacillus thuringiensis* var. israelensis formulation (strain AM65-52) against mosquitoes in mixed saltmarsh–mangrove systems and fine-scale mapping of mangrove canopy cover using affordable drone-based imagery. Pest Manag. Sci..

[14] Rumbos C.I., Athanassiou C.G. (2020). Assessment of selected larvicides for the control of *Culex pipiens* biotype pipiens and *Culex pipiens* biotype molestus under laboratory and semi-field conditions. Pest Manag. Sci..

[15] Liu T., Xie Y.G., Lin F., Xie L.H., Yang W.Q., Su X.H., Ou C.Q., Luo L., Xiao Q., Gan L. (2021). A long-lasting biological larvicide against the dengue vector mosquito *Aedes albopictus*. Pest Manag. Sci..

[16] Huang Y., Lin M., Jia M., Hu J., Zhu L. (2020). Chemical composition and larvicidal activity against Aedes mosquitoes of essential oils from *Arisaema fargesii*. Pest Manag. Sci..

[17] Milugo T.K., Tchouassi D.P., Kavishe R.A., Dinglasan R.R., Torto B. (2021). Derivatization increases mosquito larvicidal activity of the sesquiterpene lactone parthenin isolated from the invasive weed *Parthenium hysterophorus*. Pest Manag. Sci..

[18] Muturi E.J., Hay W.T., Behle R.W., Selling G.W. (2019). Amylose inclusion complexes as emulsifiers for garlic and asafoetida essential oils for mosquito control. Insects.

[19] Marina C.F., Arredondo-Jiménez J.I., Castillo A., Williams T. (1999). Sublethal effects of iridovirus disease in a mosquito. Oecologia.

[20] Becnel J.J. (2006). Transmission of viruses to mosquito larvae mediated by divalent cations. J. Invertebr. Pathol..

[21] Johnson R.M., Rasgon J.L. (2018). Densonucleosis viruses (‘densoviruses’) for mosquito and pathogen control. Curr. Opin. Insect. Sci..

[22] Vasilieva V., Lebedinets N., Gural A., Chigir T., Buchatsky L., Kuznetsova M. (1990). Examination of the preparation viroden safety for vertebrates. Mikrobiol. Zh..

[23] El-Far M., Li Y., Fédière G., Abol-Ela S., Tijssen P. (2004). Lack of infection of vertebrate cells by the densovirus from the maize worm *Mythimna loreyi* (MlDNV). Virus Res..

[24] Faisst S., Rommelaere J. (2000). Parvoviruses: From Molecular Biology to Pathology and Therapeutic Uses.

[25] Carlson J., Suchman E., Buchatsky L. (2006). Densoviruses for control and genetic manipulation of mosquitoes. Adv. Virus Res..

[26] Li J., Dong Y., Sun Y., Lai Z., Zhao Y., Liu P., Gao Y., Chen X., Gu J. (2019). A novel densovirus isolated from the asian tiger mosquito displays varied pathogenicity depending on its host species. Front. Microbiol..

[27] Liu P., Li X., Gu J., Dong Y., Liu Y., Santhosh P., Chen X. (2016). Development of non-defective recombinant densovirus vectors for microRNA delivery in the invasive vector mosquito, *Aedes albopictus*. Sci. Rep..

[28] Ferguson H.M., Ng’habi K.R., Walder T., Kadungula D., Moore S.J., Lyimo I., Russell T.L., Urassa H., Mshinda H., Killeen G.F. (2008). Establishment of a large semi-field system for experimental study of African malaria vector ecology and control in Tanzania. Malar. J..

[29] Liu P.-W., Xu J.-B., Dong Y.-Q., Chen X.-G., Gu J.-B. (2017). Use of a recombinant mosquito densovirus as a gene delivery vector for the functional analysis of genes in mosquito larvae. JoVE.

[30] Blazer V.S., Walsh H.L., Braham R.P., Smith C. (2018). Necropsy-based wild fish health assessment. JoVE.

[31] Schwartz L.D., Bickford A.A. (1986). Necropsy of chickens, turkeys, and other poultry. Vet. Clin. N. Am. Food Anim..

[32] Parkinson C.M., O’Brien A., Albers T.M., Simon M.A., Clifford C.B., Pritchett-Corning K.R. (2011). Diagnostic necropsy and selected tissue and sample collection in rats and mice. JoVE.

[33] Perumalsamy H., Jang M.J., Kim J.-R., Kadarkarai M., Ahn Y.-J. (2015). Larvicidal activity and possible mode of action of four flavonoids and two fatty acids identified in *Millettia pinnata* seed toward three mosquito species. Parasites Vectors.

[34] Ledermann J.P., Suchman E.L., Black IV W.C., Carlson J.O. (2004). Infection and pathogenicity of the mosquito densoviruses AeDNV, HeDNV, and APeDNV in *Aedes aegypti* mosquitoes (Diptera: Culicidae). J. Econ. Entomol..

[35] Hirunkanokpun S., Carlson J.O., Kittayapong P. (2008). Evaluation of mosquito densoviruses for controlling *Aedes aegypti* (Diptera: Culicidae): Variation in efficiency due to virus strain and geographic origin of mosquitoes. Am. J. Trop. Med..

[36] Zhai Y.-G., Lv X.-J., Sun X.-H., Fu S.-H., Fen Y., Tong S.-X., Wang Z.-X., Tang Q., Attoui H., Liang G.-D. (2008). Isolation and characterization of the full coding sequence of a novel densovirus from the mosquito *Culex pipiens pallens*. J. Gen. Virol..

[37] Berini F., Katz C., Gruzdev N., Casartelli M., Tettamanti G., Marinelli F. (2018). Microbial and viral chitinases: Attractive biopesticides for integrated pest management. Biotechnol. Adv..

[38] Chen C., Lee H., Stella-Wong S., Lau K., Sofian-Azirun M. (2009). Container survey of mosquito breeding sites in a university campus in Kuala Lumpur, Malaysia. Dengue Bull..

[39] Alkenani N.A. (2017). Influence of the mixtures composed of slow–release insecticide formulations against *Aedes aegypti* mosquito larvae reared in pond water. Saudi J. Biol. Sci..

[40] Kovendan K., Murugan K., Kumar A.N., Vincent S., Hwang J.-S. (2012). Bioefficacy of larvicdial and pupicidal properties of *Carica papaya* (Caricaceae) leaf extract and bacterial insecticide, spinosad, against chikungunya vector, *Aedes aegypti* (Diptera: Culicidae). Parasitol. Res..

[41] Guidi V., Lehner A., Lüthy P., Tonolla M. (2013). Dynamics of *Bacillus thuringiensis* var. israelensis and *Lysinibacillus sphaericus* spores in urban catch basins after simultaneous application against mosquito larvae. PLoS ONE.

[42] Bernal L., Dussán J. (2020). Synergistic effect of *Lysinibacillus sphaericus* and glyphosate on temephos-resistant larvae of *Aedes aegypti*. Parasites Vectors.

[43] Bravo A., Gill S.S., Soberon M. (2007). Mode of action of *Bacillus thuringiensis* Cry and Cyt toxins and their potential for insect control. Toxicon.

[44] Gu J.-B., Dong Y.-Q., Peng H.-J., Chen X.-G. (2010). A recombinant AeDNA containing the insect-specific toxin, BmK IT1, displayed an increasing pathogenicity on *Aedes albopictus*. Am. J. Trop. Med..

[45] Tetreau G., Stalinski R., Kersusan D., Veyrenc S., David J.-P., Reynaud S., Després L. (2012). Decreased toxicity of *Bacillus thuringiensis* subsp. israelensis to mosquito larvae after contact with leaf litter. Appl. Environ..

[46] Dupont C., Boisvert J. (1986). Persistence of *Bacillus thuringiensis* serovar. israelensis toxic activity in the environment and interaction with natural substrates. Water Air Soil Pollut..

[47] Sun Y., Dong Y., Li J., Lai Z., Hao Y., Liu P., Chen X., Gu J. (2019). Development of large-scale mosquito densovirus production by in vivo methods. Parasites Vectors.

[48] Agboli E., Leggewie M., Altinli M., Schnettler E. (2019). Mosquito-Specific Viruses—Transmission and Interaction. Viruses.

[49] Marcombe S., Darriet F., Agnew P., Etienne M., Yp-Tcha M.-M., Yébakima A., Corbel V. (2011). Field efficacy of new larvicide products for control of multi-resistant *Aedes aegypti* populations in Martinique (French West Indies). Am. J. Trop. Med..

[50] Thavara U., Tawatsin A., Chansang C., Asavadachanukorn P., Zaim M., Mulla M.S. (2007). Simulated field evaluation of the efficacy of two formulations of diflubenzuron, a chitin synthesis inhibitor against larvae of *Aedes aegypti* (L.) (Diptera: Culicidae) in water-storage containers. Southeast Asian J. Trop. Med. Public Health.

[51] Becker N., Petric D., Zgomba M., Boase C., Dahl C., Lane J., Kaiser A. (2003). Mosquitoes and Their Control.

[52] Margalit J., Bobroglo H. (1984). The effect of organic materials and solids in water on the persistence of *Bacillus thuringiensis* var. israelensis Serotype H-14 1. Z. Angew. Entomol..

[53] Karch S., Manzambi Z., Salaun J. (1991). Field trials with Vectolex *(Bacillus sphaericus*) and Vectobac (*Bacillus thuringiensis* (H-14)) against *Anopheles gambiae* and *Culex quinquefasciatus* breeding in Zaire. J. Am. Mosq. Control Assoc..

[54] Yang P., Carey J.R., Dowell R.V. (1994). Temperature influences on the development and demography of *Bactrocera dorsalis* (Diptera: Tephritidae) in China. Environ. Entomol..

[55] Dreyer H., Baumgärtner J. (1996). Temperature influence on cohort parameters and demographic characteristics of the two cowpea coreids *Clavigralla tomentosicollis* and *C. shadabi*. Entomol. Exp. Appl..

[56] Infante F. (2000). Development and population growth rates of *Prorops nasuta* (Hym., Bethylidae) at constant temperatures. J. Appl. Entomol.

[57] Muturi E.J., Alto B.W. (2011). Larval environmental temperature and insecticide exposure alter *Aedes aegypti* competence for arboviruses. Vector Borne Zoonotic Dis..

[58] Buckner E.A., Alto B.W., Lounibos L.P. (2016). Larval temperature–food effects on adult mosquito infection and vertical transmission of dengue-1 virus. J. Med. Entomol..

[59] Perrin A., Gosselin-Grenet A.-S., Rossignol M., Ginibre C., Scheid B., Lagneau C., Chandre F., Baldet T., Ogliastro M., Bouyer J. (2020). Variation in the susceptibility of urban Aedes mosquitoes infected with a densovirus. Sci. Rep..

[60] Bravo A., Likitvivatanavong S., Gill S.S., Soberón M. (2011). *Bacillus thuringiensis*: A story of a successful bioinsecticide. Insect Biochem. Mol. Biol..

[61] Buchatsky L. (1989). Densonucleosis of bloodsucking mosquitoes. Dis. Aquat. Org..

[62] Fediere G., Faisst G., Rommerlaere S. (2000). Epidemiology and pathology of densovirinae. Parvoviruses: From Molecular Biology to Pathology and Therapeutic Uses.

[63] Jousset F.-X., Barreau C., Boublik Y., Cornet M. (1993). A parvo-like virus persistently infecting a C6/36 clone of *Aedes albopictus* mosquito cell line and pathogenic for *Aedes aegypti* larvae. Virus Res..

