# Supplementary material for: Densovirus Oil Suspension Significantly Improves the Efficacy and Duration of Larvicidal Activity against Aedes albopictus
Source: Viruses. 2022 Feb 25;14(3):475. doi: 10.3390/v14030475 (PMC8954509; doi:10.3390/v14030475)
Supplement: Supplementary file 1 [file viruses-14-00475-s001.zip › viruses-1588944-supplementary.pdf]

viruses

MDPI

Supplementary Data

Densovirus Oil Suspension Significantly Improves the Efficacy and Duration of Larvicidal Activity against *Aedes albopictus*

Khadija Batool [1], Jie Xiao [1], Ye Xu [1], Ting Yang [2], Peiwen Tao [2], Siyu Zhao [1], Jiao Chen [2], Intikhab Alam [3], Yugu Xie [1], Jinbao Gu [1] and Xiaoguang Chen [1,*]

Rate of pupation in experimental semi-field

In 0-month sample, the ninth batch of larvae survived the longest in the AalDV-5+*Bti* treatment group; with 24% pupation rate, whereas the seventh batch receiving *Bti* oil had a 12% pupation rate. The survival rate was higher in the second batch of AalDV-5 treated samples, with a 52% pupation rate observed (Figure S1A). In 3-month sample treated with the mixed-oil samples, 17.3% of the longest surviving batch (eight batch) of larvae developed into pupae, whereas 16 % pupae were observed in the seventh batch of *Bti* oil samples and the rate of pupation increased to 56% in the second batch of larvae treated with AalDV-5 samples (Figure S1B). In 6-month sample, sixth batch receiving AalDV-5+*Bti* oil samples, 20% of larvae developed into pupae, whereas a 37.3% pupation rate was observed in the fifth batch of *Bti* oil samples. While second batch of larvae treated with AalDV-5 oil showed 60% pupae (Figure S1C). In 9-month sample, batch receiving treatment with Aa1DV-5+*Bti* oil for the longest duration (fourth batch) showed a 26% pupation rate, whereas the larvae treated with *Bti* oil (third batch) showed a 29.3% pupation rate. AalDV-5 oil treatment showed 65.3% of larvae (second batch) developing into pupae (Figure S1D). The water and blank oil control were applied to two batches of larvae with 80% -85% pupation rates in all testing groups.

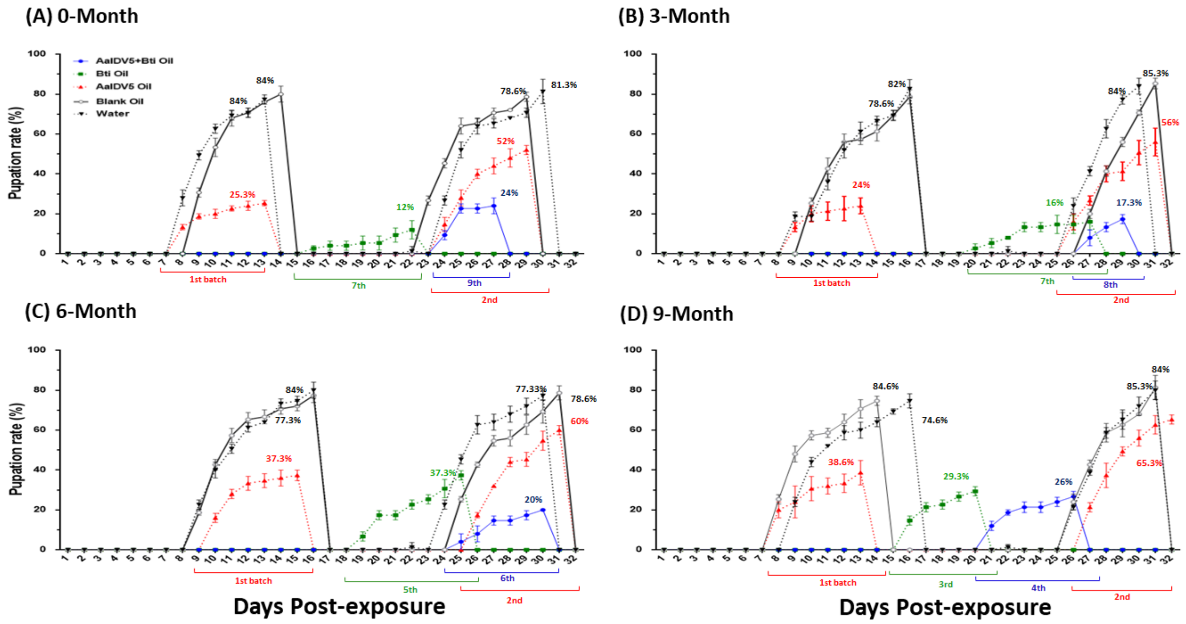

Figure S1. Rate of pupation in the semi-field bioassay. (A) Rate of pupation in the 0 month sample, (B) 3 months sample, (C) 6 months sample, (D) 9 months sample. The bars indicate SDs from three independent (biological) replicates.

Rate of pupation in experimental open-field

In 0-month sample, the AalDV-5+*Bti* treatment group, 32% of the ninth batch of larvae pupated, whereas 34.6% of the seventh batch of larvae in the *Bti* oil treatment group pupated and 57.3%

pupation rate was recorded in second batch larvae of the samples treated with AalDV-5 oil (Figure S2A). In 3-month sample, therefore, batch seven was treated for the longest duration in both the AalDV-5+*Bti* and *Bti* oil samples and 28% and 38.6% pupation rates were observed, respectively. While samples treated with AalDV-5 oil showed 60% pupation rate in second batch of larvae. (Figure S2B). In 6-month sample, more larvae survived in the fifth batch in both AalDV-5+*Bti* and *Bti* oil treatment groups, with 30.6% and 45.3% pupation rates observed, respectively and 66.6% of the larvae pupated in AalDV-5 oil treatment (Figure S2C) . In 9-month sample, the fourth batch of larvae was treated with AalDV-5+*Bti* for the longest duration, with a 29.3% pupation rate observed, whereas the third batch treated with *Bti* oil had a 32% pupation rate. Treatments with AalDV-5 oil showed 70.6% of second batch of the larvae developing into pupae (Figure S2D).

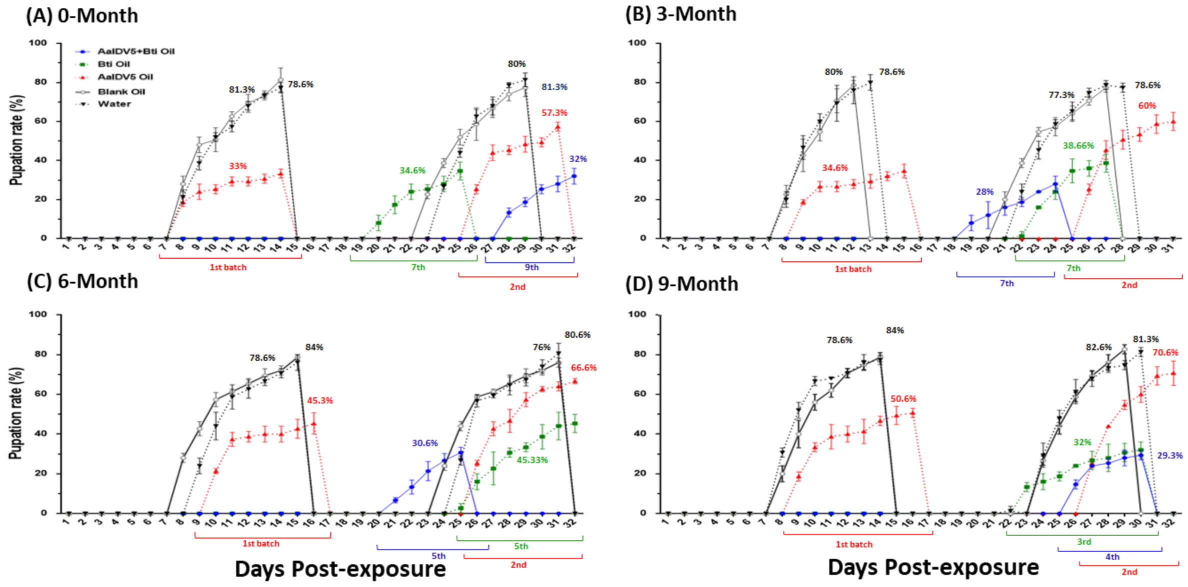

Figure S2. Rate of pupation in an open-field bioassay. (A) Rate of pupation in the 0 month sample, (B) 3 months sample, (C) 6 months sample, (D) 9 months sample. The bars indicate SDs from three independent (biological) replicates.

Table S1. The internal control used for RT-PCR and PCR.

Cell	Gene name	Forward primer	Reverse primer	Product size (bp)	Application
HBMEC	hsGADPH	GAAATGAATGGGCAGCCGTT	GAGTTAAAAGCAGCCCTGGTG	200	RT-PCR
U251	hsGADPH	GAAATGAATGGGCAGCCGTT	GAGTTAAAAGCAGCCCTGGTG	200	RT-PCR
Vero	MonkeyGAPDH	GAAATCCCATCACCATCTTCCAGG	GAGCCCCAGCCTTCTCCATG	120	RT-PCR
Cos-7	MonkeyGAPDH	GAAATCCCATCACCATCTTCCAGG	GAGCCCCAGCCTTCTCCATG	120	RT-PCR
BHK-21	MouseGAPDH	CGGCCGCATCTTCTTGTG	CCGACCTTCACCATTTTGTCTAC	299	RT-PCR
Mouse	MouseGAPDH	CGGCCGCATCTTCTTGTG	CCGACCTTCACCATTTTGTCTAC	299/450	PCR,RT.PCR
S2	S2	TACATCCCAATCCAAATCAGC	ACATCCACGCCTTCCTTG	184	RT-PCR
Sf9	SfrpL8	GTGATTCGTGCTCAGCGTAA	ACCAGGGTCATGGATGATGT	240	RT-PCR
C6/36	12RPS7	CTGATGCGTTCGAGGGTCAA	ACGCTCACCAATGAACACGA	245	RT-PCR
Aag2	AAG2	CAGGGTTCGATTCCGGAGAG	GGAAAACACTGCACCCGATG	416	RT-PCR
Cyprinus carpio	CC196	CCGACTCGGAGAGGTAGTGA	CCCGAGATCCAACTACGAGC	196	RT-PCR
Mice	(GAPDH) Acute respiratory/path.	TGATGGGTGTGAACCACGAG	GGTCATGAGCCCTTCCACAA	134	PCR
Chicken	CG450	GCGGTTCTGTGCTGTTTCAT	CGAAACTGAGCGGTGGTGAA	451	PCR,RT.PCR

Table S2. AalDV-5 titer in the larval rearing water in semi-field environment.

Sample Type	Time Interval	Day-7	Day-14	Day-21	Day-28
AalDV-5+ *Bti* Oil (copies/mL)	0 Month	6.18E+07	2.90E+07	2.63E+07	6.07E+06
	3 Months	5.84E+07	3.10E+07	2.49E+07	5.61E+06
	6 Months	5.52E+07	2.05E+07	1.13E+07	5.06E+06
	9 Months	4.99E+07	1.23E+07	5.91E+06	4.80E+06
AalDV-5 Oil (copies/mL)	0 Month	4.55E+07	9.19E+06	7.97E+06	6.08E+05
	3 Months	4.25E+07	9.18E+06	6.84E+06	4.11E+05
	6 Months	2.48E+07	8.24E+06	5.32E+06	4.64E+05
	9 Months	2.23E+07	7.00E+06	3.63E+06	2.00E+05

Table S3. AalDV-5 titer in the larval rearing water in open-field environment.

Sample Type	Time Interval	Day-7	Day-14	Day-21	Day-28
AalDV-5+ *Bti* Oil (copies/mL)	0 Month	5.28E+07	2.45E+07	1.96E+07	2.97E+06
	3 Months	4.78E+07	2.07E+07	1.41E+07	2.22E+06
	6 Months	4.20E+07	2.00E+07	1.07E+07	1.62E+06
	9 Months	2.85E+07	5.72E+06	1.55E+07	1.44E+06
AalDV-5 Oil (copies/mL)	0 Month	2.44E+07	4.15E+06	3.48E+06	1.86E+05
	3 Months	1.81E+07	3.80E+06	3.44E+06	1.58E+05
	6 Months	1.34E+07	3.67E+06	2.83E+06	1.21E+05
	9 Months	1.04E+07	3.33E+06	2.75E+06	8.44E+04

Table S4. The AalDV-5 viral accumulation in larvae in semi-field condition.

Sample Type	Time Interval	Day-7	Day-14	Day-21	Day-28
AalDV-5+ *Bti* Oil (copies/mL)	0 Month	5.01E+06	3.36E+07	6.27E+07	4.39E+07
	3 Months	4.88E+06	1.22E+07	4.75E+07	4.03E+07
	6 Months	3.59E+06	1.01E+07	3.44E+07	2.82E+07
	9 Months	2.83E+06	8.24E+06	2.95E+07	1.28E+07
AalDV-5 Oil (copies/mL)	0 Month	2.12E+06	1.56E+07	2.38E+07	1.98E+07
	3 Months	2.95E+06	7.00E+06	2.06E+07	1.61E+07
	6 Months	2.07E+06	5.48E+06	1.96E+07	1.18E+07
	9 Months	1.65E+06	4.64E+06	1.67E+07	9.86E+06

Table S5. The AalDV-5 viral accumulation in larvae in open-field condition.

Sample Type	Time Interval	Day-7	Day-14	Day-21	Day-28
AalDV-5+ *Bti* Oil (copies/mL)	0 Month	5.77E+06	1.13E+07	2.88E+07	1.02E+07
	3 Months	4.82E+06	7.93E+06	1.72E+07	9.97E+06
	6 Months	3.23E+06	7.74E+06	1.59E+07	8.70E+06
	9 Months	3.15E+06	7.24E+06	1.27E+07	4.56E+06
AalDV-5 Oil (copies/mL)	0 Month	3.67E+06	6.98E+06	1.17E+07	3.03E+06
	3 Months	3.58E+06	5.10E+06	1.09E+07	2.81E+06
	6 Months	1.96E+06	4.40E+06	1.04E+07	2.22E+06
	9 Months	1.77E+06	2.98E+06	8.26E+06	2.08E+06

Table S6. Lab strain AalDV-5 virus titer in larval rearing water and larvae body.

Time Interval	Rearing-Water (copies/mL)		Larvae-body (copies/mL)	
**Days**	**Semi-Field**	**Open-Field**	**Semi-Field**	**Open-Field**
D 1	1.00E+08	1.00E+08	8.79E+07	7.65E+07
D 2	6.84E+08	4.78E+08	8.24E+07	7.56E+07
D 3	7.87E+08	7.29E+08	3.42E+08	3.03E+08
D 4	3.62E+08	3.16E+08	4.84E+08	2.34E+08
D 5	3.02E+08	2.04E+08	2.21E+08	2.05E+08
D 6	2.14E+08	1.44E+08	1.43E+08	1.39E+08
D 7	6.67E+07	6.10E+07	1.83E+08	5.95E+07
D 9	4.43E+06	4.08E+06	4.81E+06	4.49E+06
D 11	1.26E+05	5.44E+04	3.06E+04	1.00E+05
D 13	7.33E+04	7.06E+04	5.03E+04	4.24E+04
D 15	1.24E+04	1.80E+04	2.36E+04	1.12E+04

Safety assessment of AalDV-5 on non-target species

AalDV-5 is preserved in our laboratory, and the virus is proliferated by *Ae. albopictus* larvae, extracted and purified. It was observed that after the test group and the control groups were inoculated with the virus, the cell growth status was good. After 4 days post exposure, the cells were recovered and total RNA was extracted. Further, PCR and RT-PCR was performed to detect the virus *NS1* gene. The amplification of the internal reference genes of each sample is specific. In mammals, *NS1* transcription was not detected in human cells HBMEC and U251, Vero and Cos-7 in monkey cells and BHK-21 in murine cells (Figure. S3A-i). In other insects, Drosophila S2 cells and *S. frugiperda* (Sf9) cells did not detect virus *NS1* transcription after virus inoculation. After inoculation of *Ae. albopictus* C6/36 cells and *Ae. aegypti* (Aag2) cells with the virus, transcription of the virus *NS1* were detected (Figure S3A-ii). It shows that, in vitro cell level, the mosquito densovirus can specifically infect *Ae. albopictus* and *Ae. aegypti*, but cannot infect the cells of humans, monkeys and mice in mammals, or even other insects such as fruit flies and noctuid's. Toxicity analyses of AalDV-5 on non-target species displayed the absence of toxicity in infected group of chickens, carps and mice. The results of PCR and RT-PCR showed that the gene *NS1* was not detected in different tissues (Figure S3 B-D); indicating that there was no virus replication, while the infected *Ae. albopictus* larvae virus sequences were detected at both the RNA and DNA levels in all species.

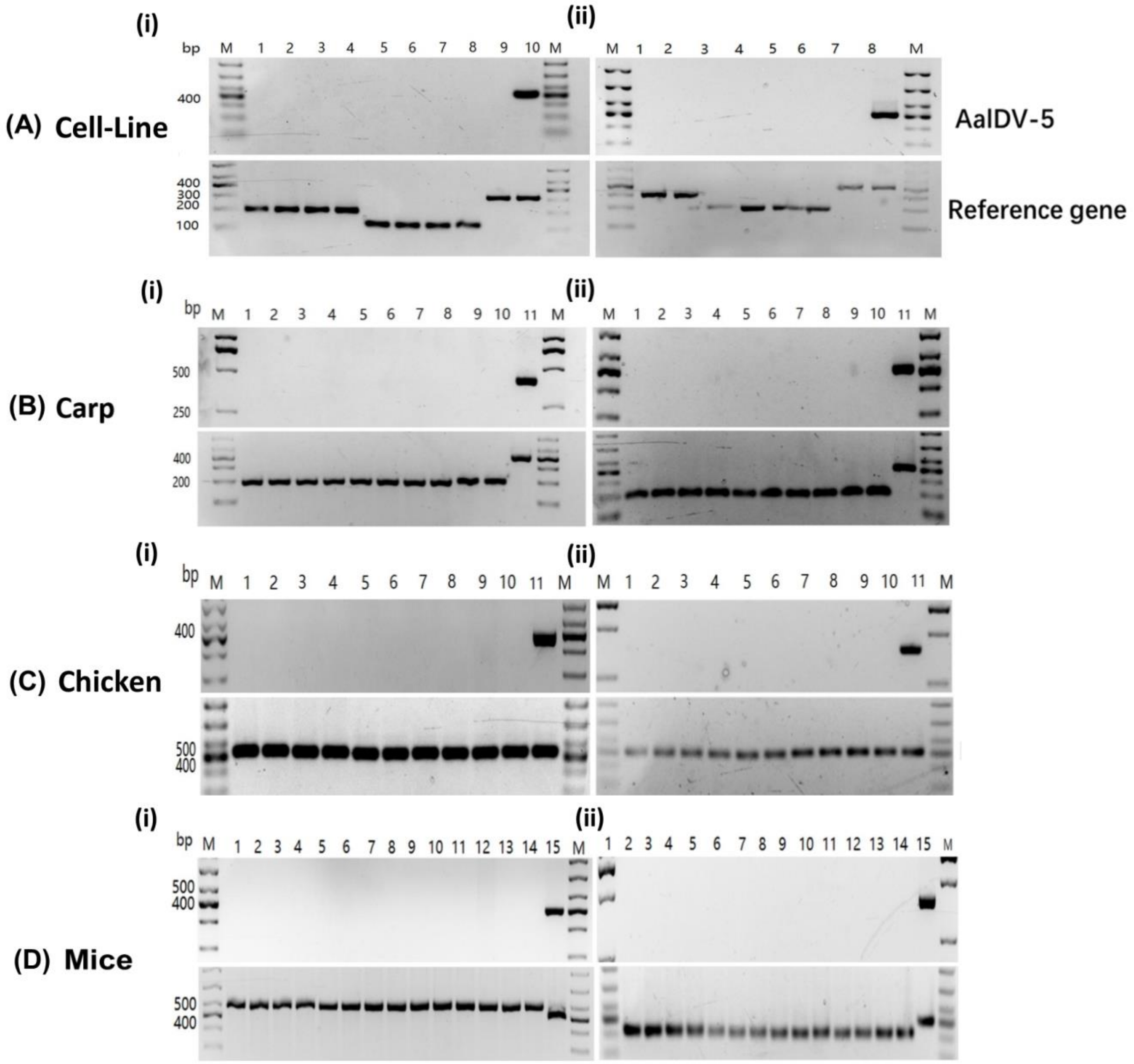

Figure S3. Effect of AalDV-5 on non- target species. **(A) Effect of AalDV-5 on in-vitro cultured cells.** i. five kinds of cells (HBMEC, U251, Vero, Cos7 and C6/36) were inoculated with AalDV-5, and the virus *NS1* was detected by RT-PCR.1. Blank group: HBMEC; 2. Infected group: HBMEC; 3. Blank group: U251; 4. Infected group: U251; 5. Blank group: Vero; 6. Infected group: Vero; 7. Blank group: Cos7; 8 Infected group: Cos7; 9. Blank group: C6/36; 10. Infected group: C6/36. (ii) Four kinds of cells (BHK-21, S2, Sf9 and Aag2) were inoculated with AalDV-5, and the mRNA level was detected by *NS1*. 1. Blank group: mouse BHK-21 cells; 2. Infected group: mouse BHK-21 cells; 3. Blank group: S2 cells; 4. Infected group: S2 cells; 5. Blank group: Sf9 Cells; 6. Infected group: Sf9 cells; 7. Blank group: Aag2 cells; 8. Infected group: Aag2 cells. **(B) Effect of AalDV-5 on Carps**. (i) PCR detection of the presence of AalDV-5 in fish tissues. 1. Infected group: gills; 2. Blank group: gills; 3. Infected group: heart; 4. Blank group: heart; 5. Infected group: swimming bladder; 6. Blank group: swim bladder; 7. Infected group: small intestine; 8. Blank group: small intestine; 9. Infected group: muscle tissue; 10. Blank group: muscle tissue; 11. Infected group: *Ae. albopictus*. (ii) RT-PCR detection of the virus *NS1* in fish tissues. 1. Infected group: gills; 2. Blank group: gills; 3. Infected group: heart; 4. Blank group: heart; 5. Infected group: swim bladder; 6. Blank group: swim bladder; 7. Infected group: small intestine; 8. Blank group: small intestine; 9. Infected group: muscle tissue; 10. Blank group: muscle tissue; 11. Infected group: *Ae. albopictus*. **(C) Effect of AalDV-5 on Chickens.** (i) PCR detection of AalDV-5 in chicken tissues 1. Infected group: heart; 2. Blank group: heart; 3. Infected group: liver; 4. Blank group: liver; 5. Infected group: lung; 6. Blank group: lung; 7. Infected group: kidney; 8. Blank group: kidney; 9. Infected group: small intestine; 10. Blank group: small intestine; 11. Infected group*: Ae. albopictus*. (ii) RT-PCR detection of AalDV-5 in chicken tissues. 1. Infected group: heart; 2. Blank group: heart; 3. Infected group: liver; 4. Blank group: liver; 5. Infected group: lungs; 6. Blank group: lung; 7. Infected group: kidney; 8. Blank group: kidney; 9.

Infected group: small intestine; 10. Blank group: small intestine; 11. Infected group: *Ae. albopictus.* **(D) Effect of AalDV-5 in Mice.** (i) PCR identification of the presence of densovirus in the mice tissues.1. Infected group: heart; 2. Blank group: heart; 3. Infected group: liver; 4. Blank group: liver; 5. Infected group: spleen; 6. Blank group: spleen; 7. Infected group: lung; 8. Blank group: lung; 9. Infection group: kidney; 10. Blank group: kidney; 11. Infection group: small intestine; 12. Blank group: small intestine; 13. Infection group: testis; 14. Blank group: testis; 15. Infected group: *Ae. albopictus*. (ii) RT-PCR detection of the virus NS1 in mice tissues.1. Infected group: heart; 2. Blank group: heart; 3. Infected group: liver; 4. Blank group: liver; 5. Infected group: spleen; 6. Blank group: spleen; 7. Infected group: lung; 8. Blank group: lung; 9. Infection group: kidney; 10. Blank group: kidney; 11. Infection group: small intestine; 12. Blank group: small intestine; 13. Infection group: testis; 14. Blank group: testis; 15. Infected group: *Ae. albopictus*.

In carps, morphological characteristics were compared between a blank control group and a treated group, and no abnormal features were detected, with both groups having normal growth and development, a good appetite, a sensitive response, a smooth body shape, coordinated movement, a balanced swimming style, a clean body, complete scales, good gloss, no tail or fin breaks, and normal weight gain (Table S7). Mice receiving the virus were able to move freely, respond quickly, and had a normal appetite, weight gain, good hair gloss, coordinated movements, a clean anus, and moderate dry feces humidity, similar to the control group, with no abnormal changes observed (Table S7). Furthermore, the chicken, carp and mice in the control group and test group were dissected, and there were no pathological changes observed. The internal organs were operating normally, the size of the internal organs was appropriate, and their color was normal.

Table S7. Effect of AalDV-5 on non-target species.

Sr. no	Specie	Test batch	Quantity	Average weight-before test (g)	Observation (days)	Health state	30-days average weight (g)
1	Chicken	1	3	72.2	30	Normal	135.6
		2	3	70.1	30	Normal	128.9
		3	3	72.6	30	Normal	131.7
		Control	3	71.8	30	Normal	133.6
2	Carp	1	3	10.1	30	Normal	10.3
		2	3	9.5	30	Normal	9.9
		3	3	10.3	30	Normal	11.1
		Control	3	9.8	30	Normal	10.9
3	Mice	1	3	25.9	30	Normal	32.3
		2	3	26.1	30	Normal	33.1
		3	3	25.8	30	Normal	34.2
		Control	3	26.6	30	Normal	32.6

Acute respiratory/ injection pathogenecity test

Acute respiratory toxicity was further assessed in mice to determine the effect of virus AalDV-5 infection through respiratory tract. The results indicated that AalDV-5 is not pathogenic to mice. The mice (both males and females) in the control group and the test group were dissected and no pathological changes were observed. Moreover, we separated the heart, liver, spleen, lung, and kidney tissues of both male and female mice, and DNA was extracted. *NS1* gene was not detected in the blank group and infected group including males after 3 day pi (Figure. S4A (i)), 1 week pe (Figure. S4A (ii)), 2 weeks pe (Figure. S4A (ii)), and 3 weeks pe (Figure. S4A (iv)). Similarly, in the females, *NS1* was not amplified after 3 days pe (Figure. S4B (i)), 1 week pe (S4B (ii)), 2 weeks pe (Figure. S4B (iii)), and 3 weeks pe (Figure. S4B (iv)). Therefore, there was no virus replication, in-

dicating that the mice were not infected (Table S8). Eight male and eight female mice were injected with the AalDV-5 virus (10^8 copies/mL) through the abdominal cavity to further detect the infection. Additionally, DNA was extracted from different tissues (heart, liver, spleen, lung, and kidney) and amplified along with specific internal reference genes respectively. The conserved viral *NS1* gene was not detected in male tissues after 3 days pe (Figure. S4C (i)), 1 week pe (Figure. S4C (ii)), 2 weeks pe (Figure. S4C (iii)), and 3 weeks pe (Figure. S4C (iv)), as well as in female tissues 3 day pe (Figure. S4D (i)), 1 week pe (Figure. S4D (ii)), 2 weeks pe (Figure. S4D (iii)), and 3 weeks pe (Figure. S4D (iv)). Furthermore, no signs of abnormal function were observed after dissection. Thus, the results confirmed that AalDV-5 caused no infection after intra-peritoneal injection of virus (Table S9).

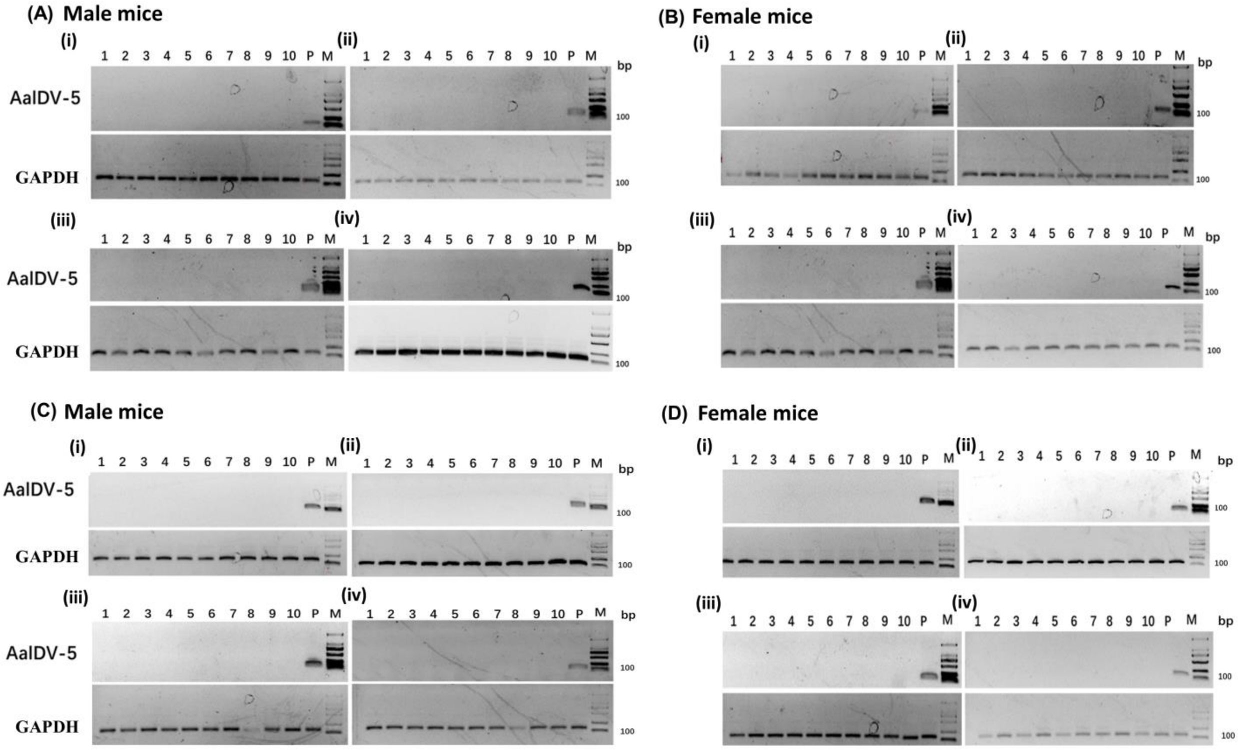

Figure S4. Acute respiratory toxicity and injection pathogenicity test in male/female mice. **(A)** PCR detection of the presence of AalDV-5 in the tissues of male mice. (i) 3 dpe (ii) 1 week pe (iii) 2 weeks pe (iv) 3 weeks pe, 1-5 Blank group: 1 heart; 2 liver; 3 spleen; 4 lungs; 5 kidney; 6-10 infection group: 6 heart, 7 liver, 8 spleen, P positive control; M marker; 9 lungs, 10 kidney **(B)** PCR detection of the presence of AalDV-5 in the tissues of female mice. (i) 3 dpe (ii) 1 week pe (iii) 2 weeks pe(iv) 3 weeks pe, 1-5 Blank group: 1 heart; 2 liver; 3 spleen; 4 lungs; 5 kidney; 6-10 infection group: 6 heart; 7 liver; 8 spleen; 9 lungs; 10 kidney; P positive control; M marker.**(C)** Acute injection toxicity test in male and female mice. The virus concentration was 10^8copies/ml, and the injection volume did not exceed 0.5-1ml/100g body weight. PCR detected the presence of AalDV-5 in the tissues of male mice. (i) 3 dpe (ii) 1 week pe (iii) 2 weeks pe (iv) 3 weeks pe in male mice, 1-5 Blank group: 1 heart; 2 liver; 3 spleen; 4 lungs; 5 kidney; 6-10 infection group: 6 heart; 7 liver; 8 spleen; 9 lungs; 10 kidney; P positive control; M marker. **(D)** PCR detection of the presence of AalDV-5 in the tissues of female mice. (i) 3 dpe (ii) 1 week pe (iii) 2 weeks pe (iv) 3 weeks pe, 1-5 Blank group: 1 heart; 2 liver; 3 spleen; 4 lungs; 5 kidney; 6-10 infection groups: 6 heart; 7 liver; 8 spleen; 9 lungs; 10 kidney; P positive control; M marker.

Table S8. Acute respiratory toxicity test in mice.

Mode of Application	3-Days							
	Sample type	Gender	Weight (g)	Body length (cm)	Abnormal behavior	Clinical Abnormalities	Gross damage	Health state
Respiratory Toxicity test	Normal Saline	Male	31.66	9.1	no	no	no	healthy
		Fe-male	34.21	9.6	no	no	no	healthy
	Densovirus	Male	36.09	10.2	no	no	no	healthy
		Fe-male	34.41	10	no	no	no	healthy
	1-Week							
	Normal Saline	Male	34.56	9.8	no	no	no	healthy
		Fe-male	39.22	9.7	no	no	no	healthy
	Densovirus	Male	36.12	9.7	no	no	no	healthy
		Fe-male	40.44	9.6	no	no	no	healthy
	2 -Week							
	Normal Saline	Male	39.51	9.6	no	no	no	healthy
		Fe-male	34.67	9.8	no	no	no	healthy
	Densovirus	Male	41.19	9.5	no	no	no	healthy
		Fe-male	32.25	9.9	no	no	no	healthy
	3 -Week							
	Normal Saline	Male	37.75	10.1	no	no	no	healthy
		Fe-male	33.3	10.3	no	no	no	healthy
	Densovirus	Male	41.82	10.6	no	no	no	healthy
		Fe-male	33.75	10.5	no	no	no	healthy

Table S9. Acute injection toxicity test in mice.

Mode of Application	3-Days							
	Sample type	Gender	Weight (g)	Body length (cm)	Abnormal behavior	Clinical Abnormalities	Gross damage	Health state
Intraperitoneal injection	Normal Saline	Male	31.58	9	no	no	no	healthy
		Fe-male	33.97	9.5	no	no	no	healthy
	Densovirus	Male	31.46	9.2	no	no	no	healthy
		Fe-male	34.33	9.4	no	no	no	healthy
	1-Week							
	Normal Saline	Male	38.87	10.1	no	no	no	healthy
		Fe-male	40.42	10.3	no	no	no	healthy
	Densovirus	Male	31.77	9.9	no	no	no	healthy
		Fe-male	40.1	10.2	no	no	no	healthy
	2 -Week							
	Normal Saline	Male	36.62	10.1	no	no	no	healthy
		Fe-male	37.17	9.9	no	no	no	healthy
	Densovirus	Male	35.4	10.3	no	no	no	healthy
		Fe-male	35.57	10.1	no	no	no	healthy
	3 -Week							
	Normal Saline	Male	42.39	10.9	no	no	no	healthy
		Fe-male	36.87	10.2	no	no	no	healthy
	Densovirus	Male	36.33	10.5	no	no	no	healthy
		Fe-male	35.39	10.5	no	no	no	healthy
